# Serotonin induced hepatic steatosis is associated with modulation of autophagy and notch signaling pathway

**DOI:** 10.1186/s12964-018-0282-6

**Published:** 2018-11-08

**Authors:** Suryakant Niture, Maxwell A. Gyamfi, Habib Kedir, Elena Arthur, Habtom Ressom, Gagan Deep, Deepak Kumar

**Affiliations:** 1 Julius L. Chambers Biomedical Biotechnology Research Institute, North Carolina Central University Durham, 1801 Fayetteville St, Durham, NC 27707 USA; 2 Department of Pharmaceutical Sciences, North Carolina Central University, Durham, NC 27707 USA; 3 Lombardi Comprehensive Cancer Center, Georgetown University Medical Center, Washington, DC 20008 USA; 4 Wake Forest Baptist Comprehensive Cancer Center, Wake Forest School of Medicine, Winston-Salem, NC 27109 USA

**Keywords:** Serotonin, Notch signaling, Autophagy, Cell steatosis, Drug resistance

## Abstract

**Background:**

Besides its neurotransmitter and vasoconstriction functions, serotonin is an important mediator of numerous biological processes in peripheral tissues including cell proliferation, steatosis, and fibrogenesis. Recent reports indicate that serotonin may promote tumor growth in liver cancer, however, the molecular mechanisms remain elusive. n this study, we investigated the role and molecular signaling mechanisms mediated by serotonin in liver cancer cell survival, drug resistance, and steatosis.

**Methods:**

Effect of serotonin on modulation of cell survival/proliferation was determined by MTT/WST1 assay. Effect of serotonin on the regulation of autophagy biomarkers and lipid/fatty acid proteins expression, AKT/mTOR and Notch signaling was evaluated by immunoblotting. The role of serotonin in normal human hepatocytes and liver cancer cell steatosis was analyzed by Oil Red O staining. The mRNA expression levels of lipid/fatty acid proteins and serotonin receptors were validated by qRT-PCR. The important roles of autophagy, Notch signaling, serotonin receptors and serotonin re-uptake proteins on serotonin-mediated cell steatosis were investigated by using selective inhibitors or antagonists. The association of peripheral serotonin, autophagy, and hepatic steatosis was also investigated using chronic EtOH fed mouse model.

**Results:**

Exposure of liver cancer cells to serotonin induced Notch signaling and autophagy, independent of AKT/mTOR pathway. Also, serotonin enhanced cancer cell proliferation/survival and drug resistance. Furthermore, serotonin treatment up-regulated the expression of lipogenic proteins and increased steatosis in liver cancer cells. Inhibition of autophagy or Notch signaling reduced serotonin-mediated cell steatosis. Treatment with serotonin receptor antagonists 5-HTr1B and 5-HTr2B reduced serotonin-mediated cell steatosis; in contrast, treatment with selective serotonin reuptake inhibitors (SSRIs) increased steatosis. In addition, mice fed with chronic EtOH resulted in increased serum serotonin levels which were associated with the induction of hepatic steatosis and autophagy.

**Conclusions:**

Serotonin regulates liver cancer cell steatosis, cells survival, and may promote liver carcinogenesis by activation of Notch signaling and autophagy.

**Electronic supplementary material:**

The online version of this article (10.1186/s12964-018-0282-6) contains supplementary material, which is available to authorized users.

## Background

Hormone serotonin (5-hydroxytryptamine) is found in a variety of organisms including humans [1–4]. Two major pools of serotonin are found in humans: brain serotonin and peripheral serotonin [5]. Peripheral serotonin is synthesized from the amino acid, tryptophan, by two unique enzymes: tryptophan hydroxylase 1 (TPH1) and aromatic acid decarboxylase (AADC), whereas, the synthesis of brain serotonin is controlled by TPH2 and AADC [6]. Only 5% serotonin is present in the brain which is synthesized by serotonergic neurons of the brainstem and the remaining 95% peripheral serotonin is produced by the enterochromaffin cells of the gut [5]. Peripheral serotonin is unable to cross the Blood Brain Barrier (BBB), and therefore, each pool of serotonin plays unique biological functions in the brain and in the peripheral organs [2].

Peripheral serotonin, on the other hand, is involved in various physiological functions and plays an important role in glucose metabolism, gluconeogenesis, and glycolysis in the liver [7–9]. Osawa et al., [10] demonstrated that mice fed with a high fat and high fructose diet with L-tryptophan developed hepatic steatosis due to an increased level of serotonin in the blood [10]. Platelet-derived serotonin has been shown to promote liver regeneration in mice after injury by acting via serotonin receptor (HTr2A and HTr2B) [11]. Thus far, seven classes of serotonin receptors (5-HTrs) have been identified, which control serotonin uptake into cells and serotonin mediated signaling in target cells [5]. 5-HTr3 is the only ligand-gated ion channel serotonin receptor, whereas, all other serotonin receptors belong to the G-protein coupled receptor superfamily [2, 5, 12]. Overexpression of 5-HTr1D, 5-HTr2B and 5-HTr7 and lower expression of 5-HTr2A and 5-HTr5 has been reported in hepatocellular carcinoma (HCC) compared with adjacent non-tumor tissue [13]. Tissue microarray and immunohistochemistry data generated from liver cancer patients suggested that expression of 5-HTr1B and 5-HTr2B are associated with cell proliferation index, and 5-HTr1B expression is correlated with the size of the liver tumor in patients [14, 15]. In hepatocellular carcinoma, serotonin treatment disturbs Axin1/β-catenin interaction, activates Wnt/β-catenin signaling leads to increased downstream genes expression such as axin 2, cyclin 1, dickoppf-1 (DDK1) and glutamine synthetase, and promotes liver cancer cell proliferation [13]. Interestingly, serotonin regulates cell signaling differentially in liver cancer cell lines. For example, treatment of serum-deprived liver Huh7 cells with serotonin promotes cell proliferation by upregulation of Forkhead box O3a (FOXO3a) transcription factor, and by increasing phosphorylation of AKT and FOXO3a, however, this mechanism is not found in serum-deprived HepG2 and Hep3B cells [16].

In the current study, we investigated the role and molecular signaling mechanisms mediated by serotonin in hepatocellular carcinoma. We demonstrate that treatment of liver cancer cells with serotonin induced autophagy, independent of the AKT/mTOR pathway. Serotonin activates Notch signaling, induces cell steatosis, increases drug resistance, and promotes cell survival. Inhibition of autophagy/Notch proteins/5-HTr1B and 5-HTr2B receptors reduced serotonin mediated cell steatosis. However, treatment of selective serotonin reuptake inhibitors (SSRIs) increased liver cancer cell steatosis. In addition, we demonstrate that mice fed with chronic EtOH had increased hepatic steatosis, which was associated with increased serum serotonin levels, suggesting that peripheral serotonin modulates alcohol induced liver cell steatosis in mice.

## Methods

### Cell culture

Liver cancer cell lines were obtained from Georgetown University (GU) Lombardi Comprehensive Cancer Center (LCCC) cell culture repository. Liver cancer cells, HepG2 and SK-Hep1, PLC/PRF5 and Hep2B2 cells were grown in DMEM medium (Invitrogen) containing 5% Fetal Bovine Serum (FBS, Access Biologicals, Vista, CA), 50 U/ml penicillin /streptomycin (Cellgro) and incubated at 37 °C in a cell culture incubator supplied with 5% CO_2_. Normal human hepatocytes and hepatocyte growth medium were obtained from Thermofisher Scientific/Gibco. Normal human hepatocytes were grown on collagen 1 coated plate (Gibco) according to the manufacturer’s protocol. All cell lines were grown for at least 24 h and after 70 to 80% of confluency, cells were used for the experiments.

### Western blot analysis

Immunoblotting analysis was performed following standard procedures as described previously [17]. The following antibodies were used for immunoblotting; anti-LC3β I/II, anti-4E-BP1, anti- phospho-S-65-4EBP1, anti-SIRT1, anti-FOXO3a, anti-cleaved Notch1(V-1744), anti-Notch2, anti-Notch3, anti-Jagged1, anti-Hes1, anti-fatty acid synthase (FAS), anti-SCD1, anti-phospho-S79-ACC, anti-ACC, anti-phospho-S473-AKT, anti-AKT, anti-phospho-S2448-mTOR, anti-mTOR and anti-GAPDH antibodies. These antibodies were obtained from Cell Signaling Technology (Danvers, MA). Anti-PPAR-γ, anti-SREBP1, and anti-Beclin1 antibodies were obtained from Santa Cruz Biotechnology (Dallas, TX), anti-p62 from BD Bioscience (San Jose, CA), anti-ATG3 and anti-β-actin from Sigma (St. Louis, MO) and anti-L-FABP1 from Abcam. All antibodies were used as per manufacturer’s suggestions.

### RNA isolation, cDNA synthesis, and RT/qPCR

HepG2 and SK-Hep1 cells were plated in 6-well plates at a density of 1 × 10^5^ cells/wells for 24 h. Cells were treated with 0.5 mM of serotonin (Sigma) for 30 h. After 30 h, cells were harvested, washed with PBS and total RNA was isolated using TRIZOL Reagent (Invitrogen, Carlsbad, CA). In other experiments, HepG2, SK-Hep1, PLC/PRF5 and Hep2B2 cells were grown for 30 h and total RNA was isolated using TRIZOL Reagent. RNA (1 μg) was reverse transcribed using a High Capacity cDNA Reverse Transcription kit (Applied Biosystems). cDNA was mixed with Power SYBR Green PCR master mix (Applied Biosystems, Carlsbad, CA) with both forward and reverse specific primers of lipid metabolic genes and serotonin receptors genes as indicated (Additional file 1: Table S1). GAPDH was amplified as an internal control. The PCR mixtures were run on a QuantStudio-3 PCR System (Applied Biosystems) using relative quantitation according to the manufacturer’s protocols.

### Cell proliferation and MTT assay

Cell proliferation was performed in 96-well plates using WST-1 assay. HepG2 and SK-Hep1 cells (1 × 10^4^ cells/well) were grown in DMEM medium for 24 h and treated with 0.1 mM, 0.2 mM and 0.5 mM serotonin for an additional 48 h. After 48 h of incubation, 10 μl WST-1 reagent was added according to manufacturer’s instructions (Roche Applied Science, Indianapolis, IN). Cell proliferation was measured by reading the plate at 450 nm using Fluostar Omega plate reader (BMG Lab tech, Cary, NC). For MTT assay, HepG2 and SK-Hep1 cells (1 × 10^4^ cells/well) were grown in 96-well plates and treated with 0.1 to 0.5 mM of serotonin or anticancer drugs, sorafenib and regorafenib (LC laboratories), with and without serotonin, as indicated in different figures for 48 h. Cells were treated with 5 μl/well MTT reagent (5 mg/ml in PBS) and incubated at 37 °C for 1 h. Cells were washed with PBS and formazan crystals were dissolved in DMSO. Cell survival was measured by reading the plate at 570 nm using the Fluostar Omega plate reader.

### Cyto-ID green fluorescence staining for autophagy determination

To determine the serotonin mediated autophagy in HepG2 and SK-Hep1 cells, cells (1 × 10^5^ cells/well) were grown on coverslips in 6-well plates and treated with 0.2 mM and 0.5 mM serotonin, or pre-treated with 4 mM 3- methyladenine (Sigma) for 8 h and further treated with 0.5 mM serotonin for 24 h. Cells were washed with PBS and then stained with Cyto-ID green fluorescence regents (Enzo Life Sciences, Plymouth Meeting, PA, USA). The Cyto-ID green fluorescence reagent was prepared in 1x assay buffer with 5% FBS and cells were treated for 30 min at 37 °C. Cells were washed with 1x assay buffer and fixed with 4% paraformaldehyde in PBS for 20 min. After washing, cells were mounted with Vectashield (Vector Lab) containing nuclear DAPI stain. Lastly, cells were observed under an Olympus BX60 fluorescent microscope and photographed.

### Oil-red O staining

HepG2 and SK-Hep1 cells (1 × 10^5^) were grown on coverslips in 6-well plates and treated with vehicle or 100 μM oleic acid (Sigma) alone or oleic acid plus 0.5 mM and 1 mM of serotonin for 24 h as indicated. After treatment, cells were washed with PBS and fixed with 4% paraformaldehyde for 10 min, gently washed with 60% isopropanol for 1 min, and stained with 0.5% Oil Red O (Sigma) staining solution (prepared in 60% isopropanol) for 30 min as described earlier [18]. Subsequently, cells were washed with ddH_2_O and counterstained with hematoxylin for 1 min. After a second wash, cells were mounted using Permount solution (Fisher Scientific) and slides were observed under an Olympus BX51 light microscope (40X objective) and photographed. The number of Oil-Red-O-stained lipid droplets present in steatotic cells (*n* = 10), treated with oleic acid alone or in a combination of oleic acid and serotonin/other drugs, were measured and plotted.

### Oil-red O staining based steatosis quantification

HepG2 and SK-Hep1 cells (1 × 10^4^/well) were grown in 96-well plates in triplicates for 16 h. Normal human hepatocytes (1 × 10^6^) were grown on collagen I coated 6-well plates for 24 h. Cells were treated with 100 μM oleic acid alone or oleic acid plus 0.5 mM of serotonin for 24 h. After Oil Red O staining, cells were lysed in 100 μl of 1x cell lysis buffer solution (Cell Signaling, Danvers, MA) for 15 min. After gentle shaking, Oil Red O stain released from steatotic cells was then transferred to another 96-well plate and the absorbance at 405 nm was measured using Fluostar Omega plate reader (BMG Lab tech, Cary, NC) as described previously [19].

### Animal care and treatment

All animal procedures were conducted in accordance with the NIH Guidelines for the Care and Use of Laboratory Animals and approved by the NCCU Institutional Animal Care and Use Committee (IACUC). Male C57BL/6 J mice (eight to ten weeks of age) were pair-fed control diets (*n* = 4) or a standard Lieber-Decarli liquid diet, containing 5% EtOH (n = 4) (representing 27.5% of the total caloric intake), for 8 weeks. The EtOH-fed group of mice was allowed free access to EtOH-containing diets with increasing concentrations of EtOH (1–5%) over a 7-day period as previously described [20]. The EtOH concentration was kept thereafter at 5% for an additional 7 weeks. EtOH comprised 27.5% of the total caloric intake of mice in this group. Liquid diets, which were purchased from a single source (DYETS Inc., Bethlehem, PA), were based upon the Lieber-DeCarli EtOH formulation and provided one kcal/mL. After 8 weeks, mice were anesthetized with isoflurane and sacrificed. Sections of liver tissue were rapidly dissected, weighed, snap-frozen in liquid nitrogen and kept at − 80 °C. Liver tissue extracts were prepared as described previously [20] and 30 (μg) protein extracts were immunoblotted with Hes1, LC3β I/II, Beclin1 and GAPDH antibodies. Liver slices were also fixed in 10% formalin/phosphate-buffered saline, and liver sections were prepared. The sections were stained with H & E for histological examination. Blood samples collected by cardiac puncture from anesthetized mice were centrifuged at 3000 rpm for 15 min and serum samples were prepared and stored at − 80 °C. Serum samples were used for measurement of serotonin levels.

### Serotonin quantification

Serotonin ELISA Kit (Enzo Life Sciences, Inc. NY) was used to quantify serotonin concentration in HepG2 and Sk-Hep1 cell lysates as well as control and EtOH-fed mice serum samples (*n* = 4). Cell lysates and serum were diluted (1:16) in assay buffer and serotonin concentrations were determined as per the manufacturer’s instructions (Enzo Life Sciences, Inc. NY).

### Statistical analysis

The data from cell proliferation, MTT assay, and cell steatosis lipid droplet quantification and animal studies were analyzed using the two-tailed Student’s *t*-test. Data are expressed as the mean ± S.D. *p* values are shown in the figures.

## Results

### Serotonin promotes liver cancer cell survival and cell steatosis

**Fig. 1:**
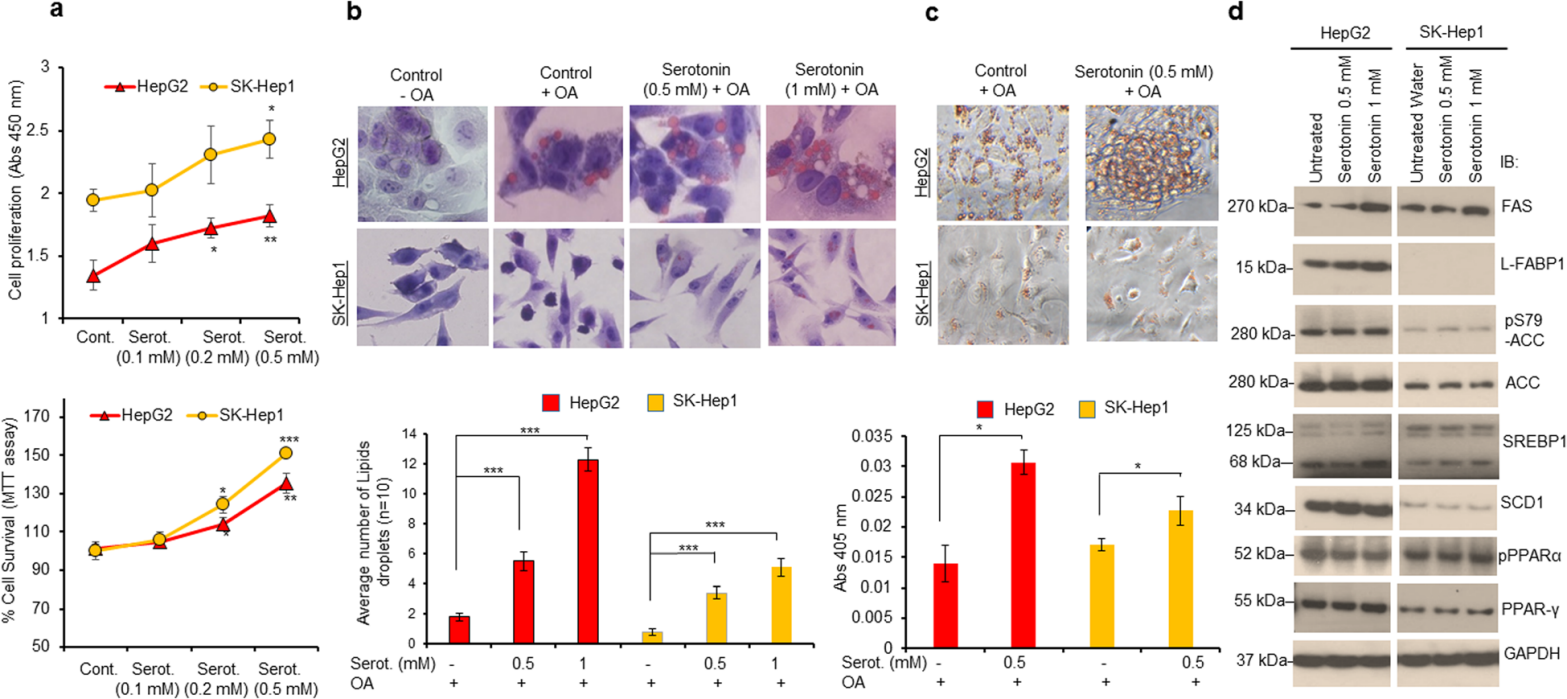
Serotonin promotes cell survival and cell steatosis in liver cancer cells HepG2 and SK-Hep1. **a** Upper Panel: HepG2 and SK-Hep1 cells were grown in 96 well plates and treated with indicated concentrations (0.1–0.5 mM) of serotonin for 48 h and cell proliferation was measured using WST1 assay as described in the materials and methods section. Lower Panel: HepG2 and SK-Hep1 cells were grown in 96 well plates treated with indicated concentrations of serotonin for 48 h. Cell survival was measured using MTT assay on Fluostar Omega plate reader as described in materials and methods section. **b** Oil-Red-O-stained lipid droplets present in HepG2 and Sk-Hep1 cells treated with different concentrations of serotonin (0.5 mM and 1 mM) were measured from the cells (*n* = 10) and plotted (upper and lower panels). **c** Oil Red O staining and representative images observed under a light microscope after oleic acid and serotonin treatment (upper panel). After Oil Red O staining, cells were lysed and Oil Red O stain released from steatotic cells was then transferred to another 96-well plate and the absorbance at 405 nm was measured as described previously [19] (lower panel). **d** HepG2 and SK-Hep1 cells were treated with indicated concentrations of serotonin for 24 h and 50 μg cell lysates were immunoblotted with indicated lipid metabolic proteins antibodies as described in the materials and methods section. Each combination of the cell line and drug concentration was set up in triplicate. Data are expressed as the mean ± S.D. *p < 0.1, **p < 0.01, *** *p < 0.001* compared to control/OA. Serot. - Serotonin. OA- Oleic acid

The role of peripheral serotonin in the modulation of human cancers, particularly liver cancer, is poorly understood. Using ELISA, we first confirmed that treatment of HepG2 and SK-Hep1 liver cancer cells with serotonin significantly increased intracellular levels of serotonin in both cell lines (data not shown). Treatment with increasing concentrations of serotonin also increased cell proliferation and cell survival in both cell lines by ~ 35% and ~ 48% respectively, compared with untreated cells (Fig. 1a, upper and lower panels). In an earlier study, hepatic steatosis was observed when mice were fed with a high fat and high fructose diet with L-tryptophan, a precursor of serotonin [10]. Therefore, in the present study, we analyzed the direct effect of serotonin on steatosis induction in normal human hepatocytes and liver cancer cells HepG2 and SK-Hep1. Oil-Red-O (ORO) staining demonstrated that no lipid droplets were observed when cells were treated with vehicle alone, whereas, treatment of oleic acid (OA) showed lipid droplet formation in cells (Fig. 1b, upper panel, Additional file 2: Figure S1). Serotonin treatment increased lipid droplet formation by 4 to 5 folds, compared with oleic acid treated cells (Fig. 1b, upper and lower panels). Serotonin treatment also induced steatosis in normal human hepatocytes (Additional file 2: Figure S1a and b). In addition, ORO-based steatosis (lipid content) quantification clearly demonstrated that serotonin treatment increased the intracellular oleic acid uptake by ~ 2 fold in HepG2 cells, ~ 1.41 fold in SK-Hep1 cells, and ~ 1.32 fold in normal human hepatocytes, compared with oleic acid alone treated cells (Fig. 1c, upper and lower panels and Additional file 2: Figure S1b).

Since numerous lipid and fatty acid metabolic enzymes/proteins regulate cell steatosis [21], we analyzed the effect of serotonin on the expression of lipid/fatty acid metabolic proteins, including fatty acid synthase (FAS), liver fatty acid-binding protein-1 (L-FABP1), acetyl-CoA carboxylase, (Acc), stearoyl-CoA desaturase-1 (SCD1), and transcription factors such as sterol regulatory element-binding protein 1 (SREBP1), peroxisome proliferator-activated receptor alpha and gamma (PPARα and PPARγ). Immunoblotting data demonstrated serotonin mediated induction in the expression of FAS, L-FABP1, ACC and SCD1 proteins by 1.5 to 2-fold in HepG2 cells. Serotonin treatment also increased the expression of transcription factors SREBP1, PPARγ, and PPARα (Fig. 1d, left panel). In SK-Hep1 cells, serotonin increased the expression of FAS, PPARγ, and PPAR-α by 1.3 to 1.5 fold, compared with untreated cells (Fig. 1d, right panel). Interestingly, the expression of L-FABP1 was not detected in SK-Hep1 cells (Fig. 1d, right panel). RT/qPCR data suggest that treatment with serotonin increased the expression of L-FABP1 and PPARγ by 1.4 and 1.7 fold compared with control HepG2 cells, whereas, expression of FAS, ACC, and SREBP1 was downregulated. (Additional file 3: Figure S2a). No significant change in the expression of mRNA levels for PPARγ, FAS, ACC, SCD1, SREBP1, and HNF4a was noted in SK-Hep1 cells (Additional file 3: Figure S2b). The data presented in Fig. 1 suggests that serotonin treatment increased liver cancer cell survival and steatosis associated with the modulation of lipid metabolic enzymes/proteins.

### Serotonin induces autophagy and steatosis in liver cancer cells

**Fig. 2:**
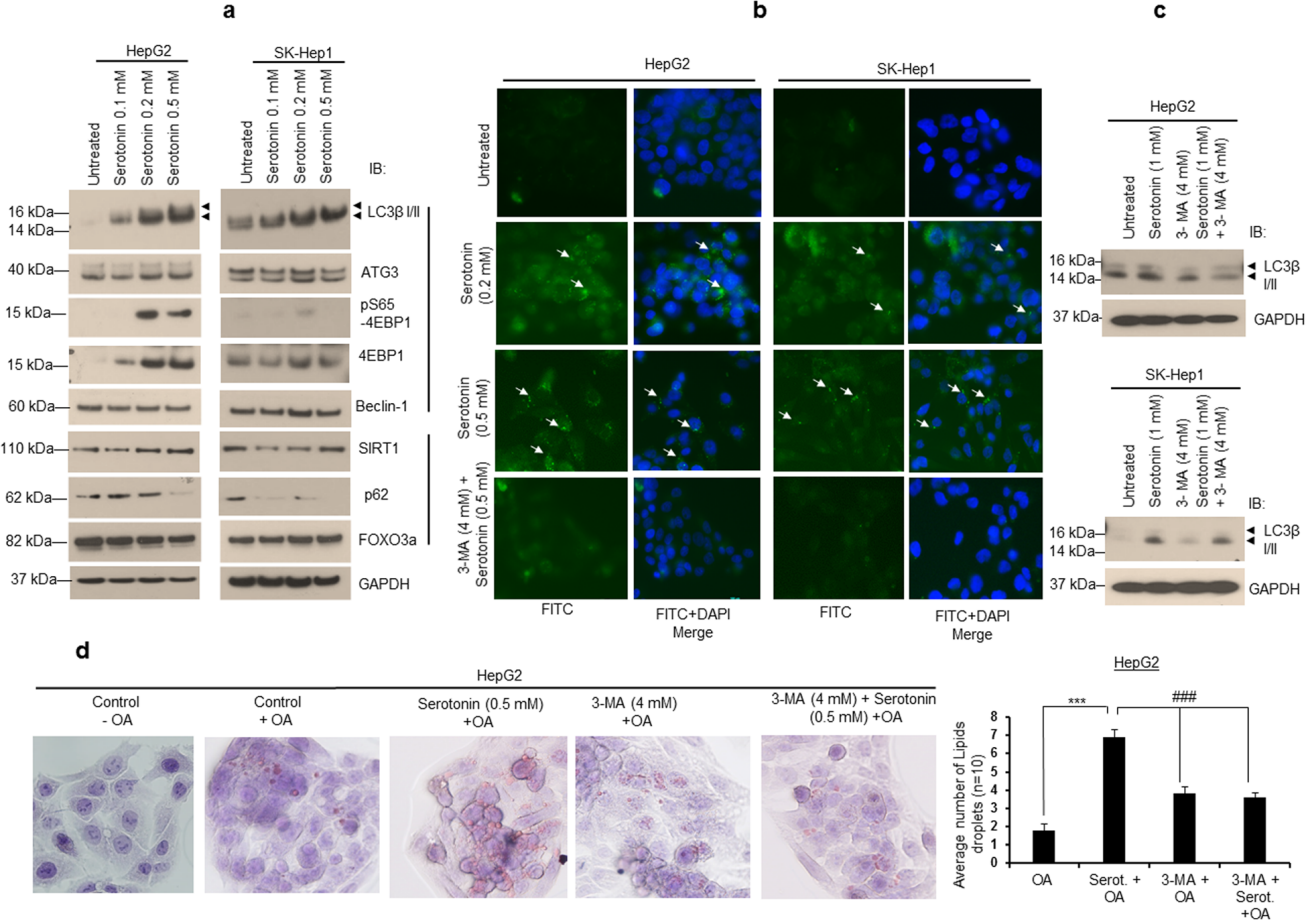
Serotonin induces autophagy. **a** HepG2 and SK-Hep1 cells were treated with indicated concentrations of serotonin for 24 h. Cells were lysed and 50 μg proteins were immunoblotted with indicated antibodies. **b** HepG2 and SK-Hep1 cells were grown on coverslips in 6-well plates and treated with 0.2 and 0.5 mM serotonin, or pre-treated with 3-MA for 8 h followed by 0.5 mM serotonin for 24 h. Cells were stained with Cyto-ID green fluorescence reagent as to determine autophagy. Cells were observed under an OlympusBX60 fluorescent microscope and photographed. **c** HepG2 and SK-Hep1 cells were grown in 6-well plates and treated with serotonin or 3-MA alone for 30 h, or pre-treated with 3-MA for 8 h followed by serotonin for 24 h as indicated, and cell lysates immunoblotted with anti-LC3β I/II and anti-GAPDH antibodies. **d** HepG2 cells were grown on coverslips in 6-well plates and treated with serotonin or 3-MA alone for 30 h or pre-treated with 3-MA for 8 h followed by serotonin treatment for 24 h as indicated. Cells were further treated with 100 μM OA for 24 h and stained with Oil Red O stain, and slides were observed under a light microscope using 40X objective and photographed (left panel). The number of Oil-Red-O-stained lipid droplets present in the steatotic cells (*n* = 10) were measured and plotted (right panel). Data are expressed as the mean ± S.D. *** *p < 0.001* compared to OA; ^# # #^ *p < 0.001* compared to Serot. 3-MA- 3-Methyladenine. Serot. - Serotonin. OA - Oleic acid

In nonalcoholic fatty liver disease (NAFLD), autophagy has been reported to play a controversial role in lipid metabolism, particularly in lipolytic and lipogenic mechanisms [22–25]. To examine whether autophagy modulates serotonin-mediated cell steatosis, we treated cells with serotonin and expression of autophagy-related proteins were analyzed by immunoblotting (Fig. 2a). Dose-dependent treatment with serotonin in HepG2 and SK-Hep1 cells induced the expression of autophagy biomarker LC3β isoform II in both cell lines (2 to 3 fold) when compared with control cells, and also increased the expression of autophagy-related effectors such as Beclin-1, ATG3, 4EBP1 and S65 phosphorylation of 4EBP1 (Fig. 2a). Serotonin treatment also increased the expression of a known autophagy-related protein SIRT1 but not p62 and FOXO3a (Fig. 2a). Furthermore, treatment with serotonin increased cytoplasmic LC3β I/II-related Cyto-ID green fluorescence in both HepG2 and SK-Hep1 cells indicating autophagy (Fig. 2b). Pre-treatment with autophagy inhibitor 3-methyladenine (3-MA), followed by serotonin treatment reduced the cytoplasmic green fluorescence (Fig. 2b) and LC3β I/II protein expression in both cell lines (Fig. 2c), suggesting that serotonin was involved in the modulation of cellular autophagy.

To test if autophagy regulates cell steatosis, HepG2 cells were treated with OA and 3-MA alone or 3-MA and serotonin. Treatment with 3-MA alone or pre-treatment with 3-MA and serotonin reduced cell steatosis. A smaller size lipid droplets and reduction in number of lipid droplets were observed when cells were exposed to 3-MA or 3-MA and serotonin compared with serotonin treatment alone (Fig. 2d, left and right panels). Together the data suggested that serotonin-mediated induction of autophagy is required for steatosis in HepG2 cells.

### Serotonin induces autophagy independent of AKT/mTOR pathway

**Fig. 3:**
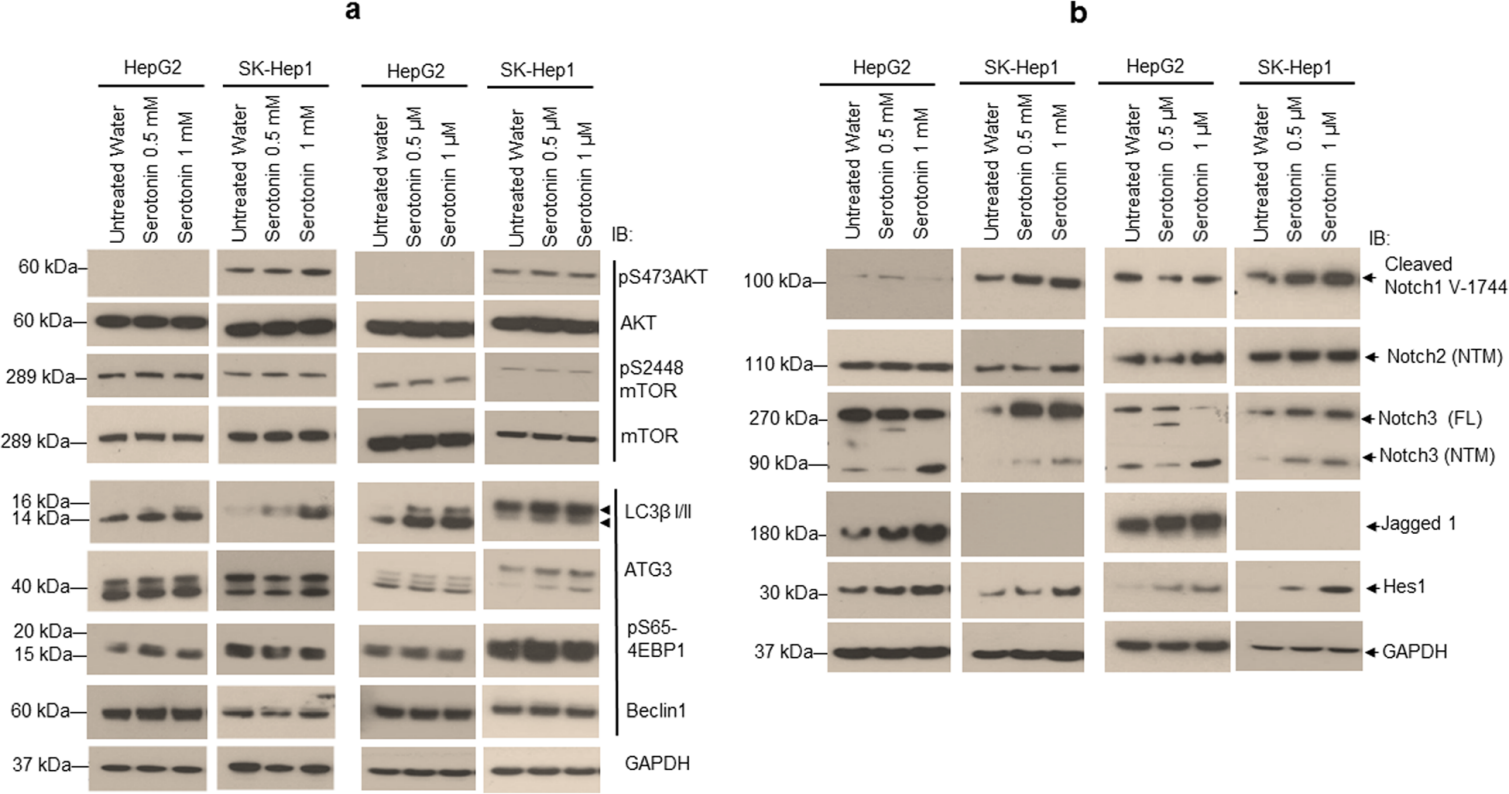
Serotonin induces AKT/mTOR-independent autophagy and activates Notch signaling. **a** HepG2 and SK-Hep1 cells were treated with high (0.5 and 1 mM) and low (0.5 and 1 μM) concentrations of serotonin for 24 h. Cells were lysed and 50 μg proteins were immunoblotted with anti-phospho-Serine-473-AKT, anti-total AKT, anti-phospho-Serine-2448-mTOR, anti-total mTOR, anti-LC3β I/II, anti-ATG3, anti-phospho-Serine-65-4EBP1, anti-Beclin1 and anti-GAPDH antibodies. **b** HepG2 and SK-Hep1 cells were treated with indicated concentrations of serotonin for 24 h. Cells were washed with PBS, lysed with cell lysis buffer and 50 μg proteins were immunoblotted with anti-cleaved-Notch1-V1744, anti-Notch2, anti-Notch3, anti-Jagged1, anti-Hes1 and anti-GAPDH antibodies

In cancer cells, AKT/mTOR pathway controls cellular autophagy [26, 27], therefore, we tested the effects of serotonin on AKT/mTOR signaling and autophagy. Using immunoblotting, we demonstrate that AKT-Ser-473 phosphorylation was not detected in HepG2 cells, and no change in phosphorylation of AKT-Ser-473 was observed in SK-Hep1 cells when cells were treated with high or low concentrations of serotonin (Fig. 3a). Similarly, no change in phosphorylation of Ser-2448 of mTOR was observed when HepG2 and SK-Hep1 cells were treated with high or low concentrations of serotonin (Fig. 3a, left and right panels). Both concentrations of serotonin treatment increased the levels of autophagy markers such as LC3βI/II, ATG3, Beclin-1 and pS65-4EBP1 (Fig. 3a).

### Serotonin induces notch signaling in liver cancer cells

Next, we examined the role of serotonin in the regulation of oncogenic Notch signaling, which is constitutively active in HCC [28–30]. Immunoblotting data indicate that treatment with high or low concentrations of serotonin did not show any change in cleaved Notch1 (cNotch1-V-1744) expression in HepG2 cells, whereas, cNotch1 protein expression was increased significantly in SK-Hep1 cells (Fig. 3b). Moreover, treatment with serotonin increased the expression of transmembrane/intracellular region (NTM) domains of Notch2 and Notch3 in both cell lines (Fig. 3b). Activation of Notch signaling requires the engagement of the Notch receptor and its ligand, DSL protein Jagged1 [28]; and our results clearly demonstrate that serotonin treatment significantly enhanced the expression of Jagged1 (2 to 4 fold) in HepG2 cells, but was not detected in SK-Hep1 cells (Fig. 3b). Finally, serotonin-mediated activation of Notch signaling was confirmed by analysis of Notch downstream expression of the transcription factor, hairy and enhancer of split-1 (Hes1), in both cell lines (Fig. 3b). The data clearly demonstrated that serotonin treatment increased the expression of Notch target Hes1 in both cell lines by 3 to 4 fold compared with control cells suggesting that serotonin activates the Notch signaling pathway in liver cancer cells.

### Inactivation of notch signaling reduces serotonin-mediated cellular autophagy and steatosis

**Fig. 4:**
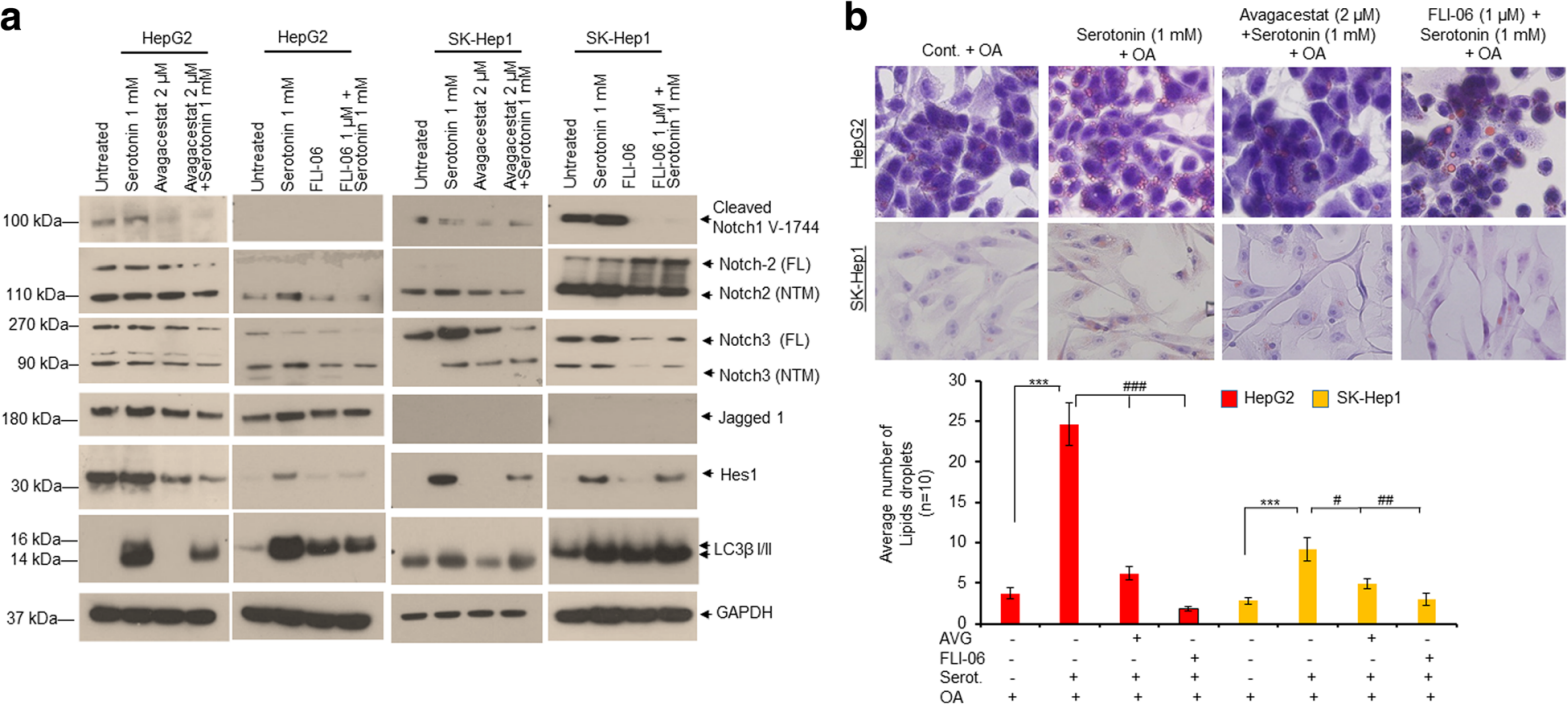
Inactivation of Notch signaling reduces cell steatosis in liver cancer cells HepG2 and SK-Hep1. **a** HepG2 and SK-Hep1 cells were treated with 1 mM serotonin alone and in combination with Notch inhibitors, 2 μM AVG and 1 μM FLI-06 as indicated for 24 h. Fifty micrograms of cell extracts were immunoblotted with anti-cleaved-Notch1V1744, anti-Notch2, anti-Notch3, anti-Jagged1, anti-Hes1, anti-LC3β I/II and anti-GAPDH antibodies. **b** HepG2 and SK-Hep1 cells were grown on coverslips in 6-well plates and treated with 1 mM serotonin alone and in combination with Notch inhibitors, 2 μM AVG and 1 μM FLI-06 as indicated for 24 h. Cells were further treated with 100 μM OA for another 24 h and stained with Oil Red O stain. Slides were observed under a light microscope using 40X objective and photographed (upper panel). The number of Oil-Red-O stained lipid droplets present in steatotic cells (*n* = 10) were measured and plotted (lower panel). Data are expressed as the mean ± S.D. *** *p < 0.001* compared to control OA; ^*#*^ *p < 0.1,* ^# #^ *p < 0.01,* ^# # #^ *p < 0.001* compared to Serot. AVG- avagacestat. Serot. - Serotonin. OA- Oleic acid

In order to determine whether Notch signaling regulates serotonin-mediated autophagy and cell steatosis, we tested the effects of two specific Notch inhibitors, avagacestat (AVG) (γ-secretase inhibitor) and FLI-06 (which acts upstream of α and β-secretase to block the intracellular trafficking of Notch signaling pathway), on regulation of Notch signaling, autophagy biomarker LC3β I/II and cell steatosis in HepG2 and SK-Hep1 cells. Immunoblotting data demonstrate that treatment with Notch inhibitors, avagacestat, and FLI-06, not only reduced the expression of Notch and Notch target Hes1 but also reduced the expression of autophagy biomarker LC3β I/II, compared with serotonin treated cells (Fig. 4a). In addition, cell steatosis data indicate that pre-treatment with Notch inhibitors significantly reduced serotonin-mediated cell steatosis compared with serotonin treatment alone (Fig. 4b, upper and lower panels), indicating that Notch signaling is involved in serotonin-mediated steatosis.

### Inhibition of 5-HTr1B and 5-HTr2B decreased cell steatosis, whereas, treatment with SSRIs induced serotonin-mediated cell steatosis in HepG2 cells

**Fig. 5:**
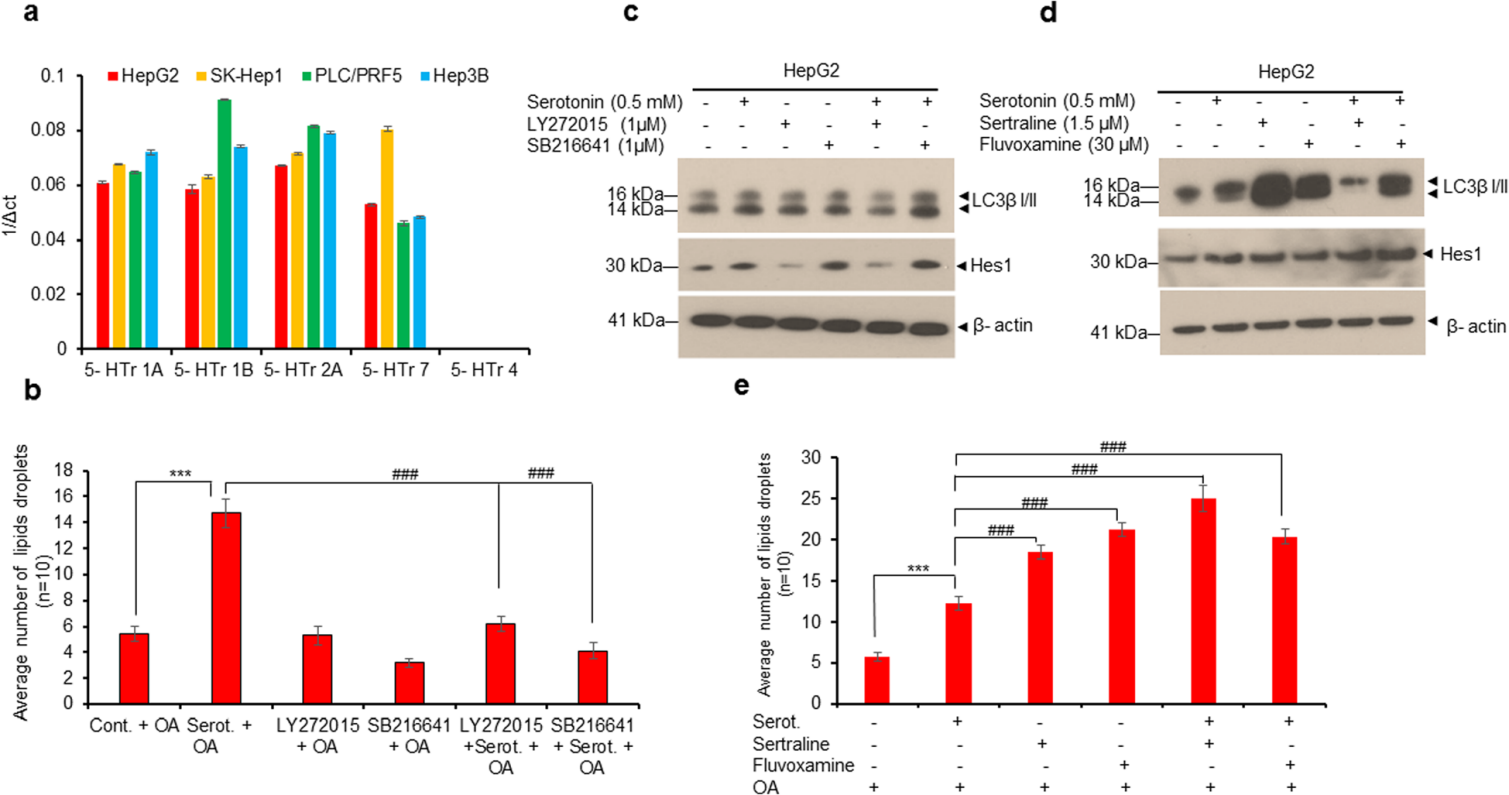
Serotonin receptors and serotonin reuptake inhibitors (SSRIs) modulate cell steatosis in liver cancer HepG2 cells. **a** Expression of various serotonin receptors (5-HTrs) in HepG2, SK-Hep1, PLC/PRF5 and Hep3B2 cells were analyzed by RT/qPCR as described in the materials and methods section. **b** HepG2 cells were grown on coverslips in 6-well plates and treated with serotonin (0.5 mM) or serotonin receptors antagonists, LY272015 (1 μM) and SB216641 (1 μM) (Abcam), alone for 30 h or pretreated with LY272015 and SB216641 for 8 h followed by serotonin treatment for 24 h in presence of LY272015 and SB216641 as indicated. Cells were further treated with vehicle alone or 100 μM OA for an additional 24 h. Cells were fixed, stained with Oil Red O stain and observed under a light microscope using 40X objective and photographed. The number of Oil-Red-O-stained lipid droplets present in each steatotic cell (n = 10) were measured and plotted. **c** HepG2 cells were grown in 6-well plates and treated with indicated concentrations of serotonin or serotonin receptors antagonists, LY272015 and SB216641 alone for 30 h, or pretreated with LY272015 and SB216641 for 8 h followed by serotonin treatment for 24 h in the presence of LY272015 and SB216641 as indicated. Fifty micrograms of cell lysates were immunoblotted with indicated antibodies. **d** HepG2 cells were treated with indicated concentrations of serotonin or serotonin re-uptake inhibitors (SSRIs), sertraline and fluvoxamine, alone for 30 h, or pretreated with sertraline and fluvoxamine for 8 h followed by serotonin treatment for 24 h in presence of SSRIs as indicated. Fifty micrograms of cell lysates were immunoblotted with indicated antibodies. **e** HepG2 cells were grown on coverslips in 6-well plates treated with indicated concentrations of serotonin or SSRIs as indicated in section 5d. Cells were further treated with vehicle alone or 100 μM OA for an additional 18 h. Cells were fixed, stained with Oil Red O stain and observed under a light microscope as described earlier. The number of Oil-Red-O-stained lipid droplets present in steatotic cells (*n* = 10) were measured and plotted. Data are expressed as the mean ± S.D. ***p < 0.001 compared to OA. ^# # #^ *p < 0.001* compared to Serot. Serot. - Serotonin. OA- Oleic acid

Serotonin receptors play an important role in serotonin uptake and liver cancer cell proliferation [13–15]. We analyzed the expression of five serotonin receptors (5-HTrs) in HepG2, SK-Hep1, PLC/PRF5 and Hep3B liver cancer cell lines by RT/qPCR (Fig. 5a). We confirmed the expression of 5-HTr1A, 5-HTr1B, 5-HTr2A, 5-HTr7, whereas, 5-HTr4 was not detected in all cell lines, as previously reported [15] (Fig. 5a). Since 5-HTr1B and 5-HTr2B are involved in liver cancer cell proliferation/tumor progression [15], we blocked the activity of 5-HTr1B and 5-HTr2B by selective antagonists, SB216641 and LY272025, respectively, and examined serotonin-mediated cell steatosis and Notch signaling (Fig. 5b, c and Additional file 4: Figure S3). Cell steatosis data demonstrate that treatment with antagonists, SB216641 and LY272025, decreased serotonin-mediated cell steatosis in HepG2 cells (Fig. 5b and Additional file 4: Figure S3). Furthermore, antagonist LY272025 blocked the serotonin-mediated activation of Notch signaling (Hes1 expression) and autophagy (LC3β I/II expression) suggesting that 5-HTr2B might be involved in serotonin-mediated Notch activation (Fig. 5c). On the other hand, treatment with serotonin reuptake inhibitors (SSRIs), sertraline and fluvoxamine, activated Notch signaling, autophagy and induced cell steatosis in HepG2 cells (Fig. 5d, e and Additional file 5: Figure S4). Interestingly, co-treatment with serotonin and sertraline resulted in reduced autophagy (Fig. 5d). Collectively, these data suggest that serotonin receptors and SSRIs may play important roles in serotonin-mediated cell steatosis in liver cancer cells and warrant further investigation.

### Serotonin increases cell survival by activation of autophagy and notch signaling

**Fig. 6:**
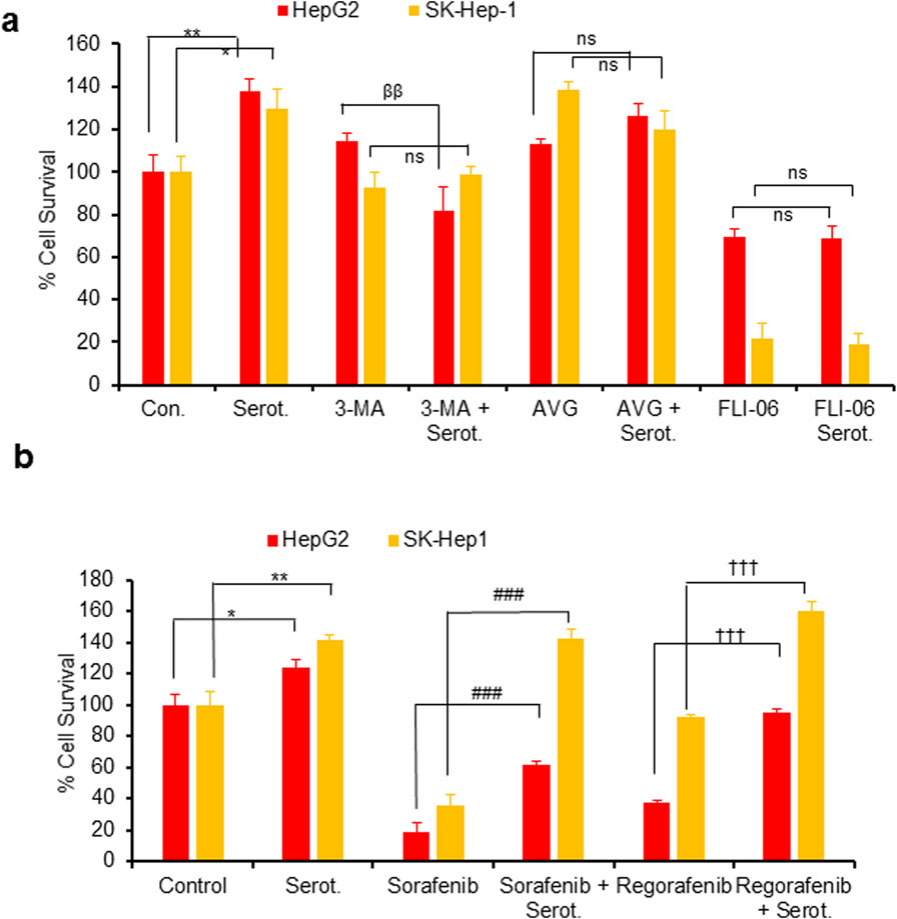
Inactivation of autophagy or Notch signaling reduces serotonin mediated cell survival while increasing serotonin induced drug resistance in HepG2 and SK-Hep1 cells. **a** HepG2 and SK-Hep1 cells were grown in 96 well plates treated with serotonin (0.5 mM), 3-MA (4 mM), serotonin plus 3-MA, avagacestat (2 μM), serotonin plus avagacestat, FLI-06 (1 μM), and serotonin plus FLI-06 as indicated for 48 h. **b** Similarly, HepG2 and SK-Hep1 cells were grown in 96-well plates and treated with serotonin (0.5 mM), sorafenib (2.5 μM), serotonin plus sorafenib, regorafenib (0.5 μM), and regorafenib plus serotonin as indicated for 48 h. Cells were treated with 5 μl MTT reagent/well (5 mg/ml in PBS) and cell survival was measured by reading the plate at 570 nm using a Fluostar Omega plate reader. Each combination of the cell line and drug concentration was set up in triplicate. Data are expressed as the mean ± S.D. * *p < 0.1,* **p < 0.01 compared to control. ^ββ^*P < 0.01* compared to 3-MA. ^###^ *p < 0.001* compared to sorafenib treatment. ^†††^*p < 0.001* compared to regorafenib treatment. Serot.-Serotonin. AVG- Avagacestat. 3-MA- 3-Methyladenine. OA- Oleic acid

The biological significance of the serotonin-mediated induction of autophagy and activation of Notch signaling in liver cancer cell survival was further investigated. Treatment of HepG2 and SK-Hep1 cells with serotonin increased cell survival by 35 to 40%; however, serotonin was unable to enhance cell survival when cells were pre-treated with autophagy inhibitor 3-MA, or Notch inhibitors, Ivagacestat and FLI-06, compared with serotonin-only-treated cells (Fig. 6a) suggesting a critical role of autophagy and Notch signaling in serotonin-mediated cell survival.

Next, we examined the effect of FDA-approved liver cancer drugs, sorafenib and regorafenib (VEGRF1–3 kinase inhibitors), on serotonin-mediated cell survival (Fig. 6b). Treatment with sorafenib decreased cell survival by 80% and 65% in HepG2 and SK-Hep1 cells, respectively (Fig. 6b). Interestingly, treatment with sorafenib along with serotonin increased cell survival by 40% in HepG2 and 80% in the SK-Hep1 cells, compared with sorafenib treatment alone. Treatment with regorafenib inhibited 60% of cell survival in HepG2 cells but had no effect on SK-Hep1 cell survival when compared with control cells (Fig. 6b). Treatment with regorafenib and serotonin increased cell survival in HepG2 by~ 40%, compared with regorafenib treatment alone, suggesting that serotonin increased drug resistance and promoted cell survival in liver cancer cells.

### Ethanol (EtOH) increases serotonin levels in mice serum and induces lipid accumulation in mice liver

**Fig. 7:**
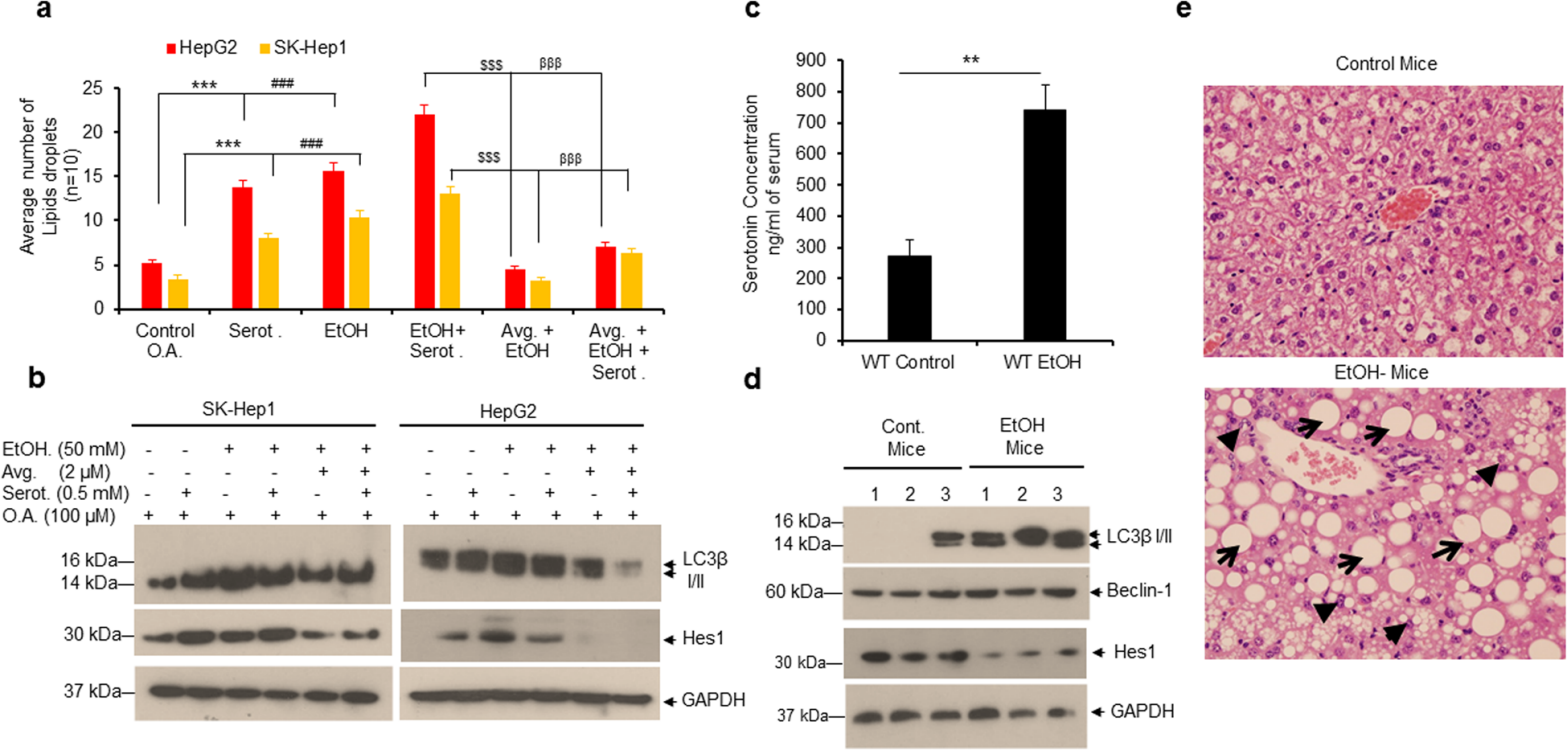
Ethanol induces lipid accumulation in both liver cancer cells and in mice fed with EtOH. **a** HepG2 and SK-Hep1 cells were grown on coverslips in 6-well plates and treated with serotonin (0.5 mM), EtOH (50 mM), EtOH + serotonin, or in combination with Notch inhibitor AVG (2 μM) + EtOH or AVG + EtOH + Serotonin as indicated for 24 h. Cells were further treated with vehicle alone or 100 μM OA for 24 h and stained with Oil Red O. The number of Oil-Red-O-stained lipid droplets present in steatotic cells (n = 10) were measured and plotted. **b** HepG2 and SK-Hep1 cells were treated as indicated in section 7a and lysates were immunoblotted with indicated antibodies. **c** Serotonin concentrations in serum samples of control and EtOH-fed mice (*n* = 4) were quantified as described in the materials and methods section. **d** Thirty micrograms of protein extracts from control and EtOH-fed mouse liver tissue were immunoblotted with indicated antibodies. **e** Representative hematoxylin and eosin (H&E)-stained liver sections of male C57BL/6 J mice pair-fed control diets (upper panel) or chronic EtOH (lower panel) (original magnification 400X). Macrovesicular (indicated by arrows) and microvesicular steatosis (indicated by arrowheads) were observed only in EtOH-fed mouse livers. Data are expressed as the mean ± S.D. **p < 0.01 compared to mice fed with control diet. ***p < 0.001, compared to OA. ^###^*p < 0.001* compared to serot. ^$$$^*p < 0.001* compared to EtOH + serot. ^βββ^*p < 0.001* compared to AVG + EtOH. AVG- avagacestat. EtOH- Ethanol. Serot.- Serotonin. OA- Oleic acid

Excessive consumption of alcohol can cause hepatic steatosis (fatty liver), which can progress to fibrosis, cirrhosis, and liver cancer [20, 31, 32]. We investigated the effects of EtOH on serotonin-mediated liver cancer cell steatosis, autophagy and Notch signaling in human liver cancer cells HepG2 and SK-Hep1. Our data suggests that treatment with serotonin, EtOH or serotonin in combination with EtOH induced cell steatosis, autophagy along with activation of Notch signaling in both cell lines (Fig. 7a, b and Additional file 6: Figure S5). However, pretreatment of cells with Notch inhibitor, avagacestat, significantly decreased serotonin or EtOH-mediated cell steatosis and autophagy (Fig. 7a and b). Furthermore, to confirm the involvement of peripheral serotonin in the induction of hepatic steatosis, we used an alcoholic C57BL/6 J mouse model. Male mice were pair-fed control liquid diets (control) or EtOH-containing diets for 8 weeks and serum serotonin levels were quantified by ELISA (Fig. 7c). EtOH ingestion significantly increased serum serotonin levels (2.74 fold) compared with control mice (Fig. 7c). Further, immunoblotting data demonstrated that EtOH induced autophagy in mice liver (as indicated by LC3β I/II and Beclin1 expression) but decreased Hes1 expression compared with control mice (Fig. 7d). Lastly, we observed that chronic EtOH feeding produced both macrovesicular (indicated by arrows) and microvesicular steatosis (indicated by arrowheads) in mice liver (Fig. 7e, lower panel), but not in mice fed the control diet (Fig. 7e, upper panel). The data presented in Fig. 7 suggests that EtOH ingestion increased serum serotonin levels in mice which may have induced hepatic steatosis by enhancing autophagy.

## Discussion

Peripheral serotonin produced by the enterochromaffin cells of the gut is involved in various physiological functions and plays an important role in the regulation of energy homeostasis, glucose metabolism, gluconeogenesis, glycolysis, lipid metabolism, bone density, and diseases associated with metabolic syndromes, such as obesity and type 2 diabetes. [7–9, 33, 34]. Peripheral serotonin also acts as a metabolic regulator in the development of obesity [35]. A recent study demonstrated that, under high-fat diet conditions, the inhibition of serotonin synthesis reduced body weight gain, improved glucose tolerance, increased thermogenic activity in brown adipose tissue and decreased lipogenesis in white adipose tissue in mice, indicating that adipocyte-derived serotonin plays important roles in controlling energy homeostasis, obesity and metabolic disease [36]. The role of peripheral serotonin in human social behavior remains unclear. An opposite correlation of plasma serotonin levels was found in men and women which are in love and not in love [37], and elevated serotonin levels were observed in older women [38]. Peripheral serotonin levels are also correlated with various human disease conditions, for example, increased levels of serotonin in blood plasma were observed in cigarette smokers [39], patients with coronary artery disease [40] and heart failure patients [41], whereas decreased free and total serum tryptophan (a serotonin precursor) in serum and serotonin levels in whole blood were found in people after acute ethanol consumption [42, 43]. Also, a reduction in platelet level serotonin was observed in Alzheimer’s patients [44].

Several studies have demonstrated that peripheral serotonin is also involved in liver cancer progression. Increased serum and plasma serotonin levels were found in HCC patients [45]. Serum serotonin levels are also higher in cirrhotic patients compared with chronic hepatitis, and cirrhotic patients with HCC compared with and cirrhotic patients without HCC [46]. The molecular mechanisms how peripheral serotonin could modulate liver cancer progression remains unknown. In the current study, we analyzed the effects of serotonin on liver cancer cell growth and survival by analyzing the key mechanisms related to liver cancer progression. Our data demonstrates that serotonin positively modulates cell proliferation/survival and cell steatosis in liver cancer cells by inducing autophagy and activating Notch signaling. We also demonstrated that mice fed with chronic EtOH showed elevated levels of serotonin in serum which is associated with increased hepatic steatosis and autophagy in mice liver.

Autophagy captures and degrades intracellular damaged organelles and misfolded or aggregated proteins through the lysosomal degradation pathway and plays a dual role in tumor growth and tumor suppression [23, 27, 47, 48]. We demonstrate that under physiological conditions, serotonin induced autophagy in both HepG2 and SK-Hep1 liver cancer cells. In contrast Soll et al., showed that serotonin inhibits starvation-induced autophagy in serum-starved Huh7 liver cancer cells [14]. In non-alcoholic fatty liver disease (NAFLD) hepatic steatosis is considered a ‘benign condition’ of liver fibrosis; which nonetheless leads to cirrhosis and ultimately HCC [20, 31, 32]. Numerous lipid and fatty acid metabolic enzymes/proteins modulate cell steatosis in liver cells [20, 21]. We show that serotonin increased the expression of several lipids and fatty acid metabolic enzymes/proteins which might be involved in liver cancer cell steatosis. A controversial role of L-tryptophan in rat hepatic steatosis has been reported previously [49, 50]. L- tryptophan alone is incapable of causing hepatic steatosis under normal diet conditions in mice, but in combination with high fat and high fructose diet, L-tryptophan induced hepatic steatosis because of the increased concentrations of peripheral serotonin [10]. Corroborating these findings, we demonstrated that mice fed with chronic EtOH showed increased hepatic cell steatosis, autophagy, and elevated levels of serotonin in mice serum, indicating that peripheral serotonin may modulate hepatic steatosis. Previous studies have also demonstrated that autophagy plays an important role in lipid metabolism and hepatic steatosis in animals [22–24]. Mice with hepatocyte-specific autophagy deficiency failed to induce fasting-mediated steatosis as shown in wild-type mice, suggesting that autophagy is essential for fasting-mediated hepatic steatosis [25].

The molecular mechanisms by which serotonin induced cell steatosis/survival and drug resistance were further investigated. Our data suggest that serotonin induced autophagy is independent of AKT/mTOR pathway, which is an important modulator of autophagy and cell survival [26, 27]. We also showed that serotonin activates Notch signaling, which is involved in liver cancer progression [28–30], and induced drug resistance in liver cancer cells. Furthermore, we demonstrated that inhibition of HTr1B and HTr2B reduced serotonin-mediated cell steatosis since these receptors are involved in liver cancer cell proliferation and tumor development [15]. On the other hand, we also demonstrated that selective serotonin reuptake inhibitors (SSRIs), which target serotonin transporter protein (SERT), increased cell steatosis in liver cancer cells. SSRIs block the function of the serotonin transporter (SERT) protein, and reduction of SERT enhances hepatic steatosis in mice [51, 52]. Reports also suggest that SERT expression is not detected in mice and human liver tissue [53, 54], however, using a SERT-specific antibody, the presence of SERT and its localization in vesicles and golgi apparatus were recently detected in HepG2 liver cancer cells [55]. The exact role of serotonin receptors, SSRIs and SERT in the regulation of serotonin-mediated liver cell steatosis and progression of carcinogenesis remains to be investigated.

Indeed, for the first time, we demonstrate that serotonin increases liver cancer cell survival/drug resistance by inducing autophagy, activating Notch signaling, and by stimulating cell steatosis in liver cancer cells. We also showed the involvement of peripheral serotonin in EtOH-induced steatosis by demonstrating that mice fed with chronic EtOH showed increased hepatic steatosis, which was associated with increased serum serotonin levels.

## Conclusions

In conclusion, our study suggests that peripheral serotonin induces autophagy independent of the AKT/mTOR pathway and activates Notch signaling. By inducing autophagy and by activation of Notch signaling serotonin induces cell steatosis, increases drug resistance, and promotes liver cancer cell survival. Inhibition of autophagy and Notch signaling reduced serotonin-mediated cell steatosis. We also demonstrate that mice fed with chronic EtOH exhibited increased hepatic steatosis and autophagy, which was associated with increased serum serotonin levels. Characterization of this signaling pathway provides a better understanding of the additional role of peripheral serotonin in the modulation of cell steatosis and liver cancer progression.

## Additional files

Additional file 1: **Table S1.** The following primer sets were used for analysis of fatty acid/lipid metabolic gene expression and serotonin receptor (5-HTrs) expression using RT/qPCR. (DOCX 15 kb)

Additional file 2: **Figure S1.** Serotonin induces cell steatosis in normal human hepatocytes. (**a**) Normal human hepatocytes were grown in 6-well plates coated with Collagen 1 (Gibco) for 24 h and treated with vehicle or 100 μM oleic acid (OA) alone, or OA and serotonin (0.5 mM) for 24 h. Cells were fixed, stained with Oil Red O stain only, and observed under using light microscopy and photographed as described earlier. (**b**) After Oil Red O staining, the same cells were lysed in cell lysis buffer (100 μl). Oil Red O stain released from steatotic cells was then transferred to another 96-well plate and the absorbance at 405 nm was measured using the Fluostar Omega plate reader as described previously [19]. **p < 0.01 compared to OA treated cells. (TIF 566 kb)

Additional file 3: **Figure S2.** Serotonin modulates the expression of fatty acid and lipid metabolic genes in HepG2 and SK-Hep1 cells. (**a**) HepG2 and (**b**) SK-Hep1 cells were grown in 6-well plates and treated with 0.5 mM of serotonin for 30 h. Total RNA was isolated from untreated and serotonin treated cells using TRIZOL reagent, and the expression of fatty acid and lipid metabolic gene expression was analyzed by RT/qPCR as described in the materials and methods sections. Data are expressed as the mean ± S.D. *p < 0.1 compared to untreated control cells. (TIF 83 kb)

Additional file 4: **Figure S3.** Effect of serotonin receptor antagonists on HepG2 cell steatosis. HepG2 cells were grown on coverslips in 6-well plates and treated with indicated concentrations of serotonin, LY272015 or SB216641 alone, or in combination, as indicated. Cells were further treated with 100 μM oleic acid for an additional 24 h. Cells were fixed, stained with Oil Red O stain, and observed under a light microscope and photographed. (TIF 508 kb)

Additional file 5: **Figure S4.** Effect of serotonin re-uptake inhibitors (SSRIs) on HepG2 cell steatosis. HepG2 cells were grown on coverslips in 6-well plates and treated with serotonin or serotonin re-uptake inhibitors (SSRIs), sertraline and fluvoxamine, alone for 30 h, or pretreated with sertraline and fluvoxamine for 8 h followed by serotonin treatment for 24 h in the presence of SSRIs as indicated. Cells were further treated with vehicle alone or 100 μM oleic acid for additional 18 h. Cells were stained with Oil Red O stain and observed under a light microscope and photographed as described earlier. (TIF 450 kb)

Additional file 6: **Figure S5.** Effect of EtOH on liver cancer cell steatosis. HepG2 and SK-Hep1 cells were grown on coverslips in 6-well plates and treated with serotonin (0.5 mM), EtOH (50 mM), or in combination with Notch inhibitor avagacestat (2 μM) as indicated for 24 h. Cells were further treated with vehicle alone or 100 μM oleic acid and stained with Oil Red O. Cells were stained with Oil Red O and observed under a light microscope and photographed. (TIF 587 kb)

## Abbreviations

3-MA: 3-Methyladenine
HCC: Hepatocellular carcinoma
LC3β I/II: Microtubule-associated proteins 1A/1B light chain 3B
MTT: (3-(4,5-dimethylthiazol-2-yl)-2,5-diphenyl tetrazolium bromide)
NAFLD: Nonalcoholic fatty liver disease
SSRIs: Selective Serotonin Reuptake Inhibitors

## References

[1] Kang K, Park S, Kim YS, Lee S (2009). Back K. biosynthesis and biotechnological production of serotonin derivatives. Appl Microbiol Biotechnol.

[2] Berger M, Gray JA, Roth BL (2009). The expanded biology of serotonin. Annu Rev Med.

[3] Curran KP, Chalasani SH (2012). Serotonin circuits and anxiety: what can invertebrates teach us?. Invertebr Neurosci.

[4] Srinivasan S, Sadegh L, Elle IC, Christensen AG, Faergeman NJ, Ashrafi K (2008). Serotonin regulates C. elegans fat and feeding through independent molecular mechanisms. Cell Metab.

[5] El-Merahbi R, Loffler M, Mayer A, Sumara G (2015). The roles of peripheral serotonin in metabolic homeostasis. FEBS Lett.

[6] Keszthelyi D, Troost FJ, Masclee AA (2009). Understanding the role of tryptophan and serotonin metabolism in gastrointestinal function. Neurogastroenterol Motil.

[7] Coelho WS, Da Silva D, Marinho-Carvalho MM, Sola-Penna M (2012). Serotonin modulates hepatic 6-phosphofructo-1-kinase in an insulin synergistic manner. Int J Biochem Cell Biol.

[8] Lin HV, Accili D (2011). Hormonal regulation of hepatic glucose production in health and disease. Cell Metab.

[9] Zabala MT, Lorenzo P, Alvarez L, Berlanga JJ, Garcia-Ruiz JP (1992). Serotonin increases the cAMP concentration and the phosphoenolpyruvate carboxykinase mRNA in rat kidney, small intestine. and liver J Cell Physiol.

[10] Osawa Y, Kanamori H, Seki E, Hoshi M, Ohtaki H, Yasuda Y, Ito H, Suetsugu A, Nagaki M, Moriwaki H, Saito K (2011). Seishima M. L-tryptophan-mediated enhancement of susceptibility to nonalcoholic fatty liver disease is dependent on the mammalian target of rapamycin. J Biol Chem.

[11] Lesurtel M, Graf R, Aleil B, Walther DJ, Tian Y, Jochum W, Gachet C, Bader M, Clavien PA (2006). Platelet-derived serotonin mediates liver regeneration. Science.

[12] Noda M, Higashida H, Aoki S, Wada K (2004). Multiple signal transduction pathways mediated by 5-HT receptors. Mol Neurobiol.

[13] Fatima S, Shi X, Lin Z, Chen GQ, Pan XH, Wu JC, Ho JW, Lee NP, Gao H, Zhang G, Lu A, Bian ZX (2016). 5-Hydroxytryptamine promotes hepatocellular carcinoma proliferation by influencing beta-catenin. Mol Oncol.

[14] Soll C, Jang JH, Riener MO, Moritz W, Wild PJ, Graf R, Clavien PA (2010). Serotonin promotes tumor growth in human hepatocellular cancer. Hepatology.

[15] Soll C, Riener MO, Oberkofler CE, Hellerbrand C, Wild PJ, DeOliveira ML, Clavien PA (2012). Expression of serotonin receptors in human hepatocellular cancer. Clin Cancer Res.

[16] Liang C, Chen W, Zhi X, Ma T, Xia X, Liu H, Zhang Q, Hu Q, Zhang Y, Bai X, Liang T (2013). Serotonin promotes the proliferation of serum-deprived hepatocellular carcinoma cells via upregulation of FOXO3a. Mol Cancer.

[17] Niture S, Ramalinga M, Kedir H, Patacsil D, Niture SS, Li J, Mani H, Suy S, Collins S, Kumar D (2018). TNFAIP8 promotes prostate cancer cell survival by inducing autophagy. Oncotarget.

[18] Zhu C, Xie P, Zhao F, Zhang L, An W, Zhan Y (2014). Mechanism of the promotion of steatotic HepG2 cell apoptosis by cholesterol. Int J Clin Exp Pathol.

[19] Cui W, Chen SL, Quantification HKQ (2010). Mechanisms of oleic acid-induced steatosis in HepG2 cells. Am J Transl Res.

[20] Choi S, Neequaye P, French SW, Gonzalez FJ, Gyamfi MA (2018). Pregnane X receptor promotes ethanol-induced hepatosteatosis in mice. J Biol Chem.

[21] Kawano Y, Cohen DE (2013). Mechanisms of hepatic triglyceride accumulation in non-alcoholic fatty liver disease. J Gastroenterol.

[22] Shibata M, Yoshimura K, Tamura H, Ueno T, Nishimura T, Inoue T, Sasaki M, Koike M, Arai H, Kominami E, Uchiyama Y (2010). LC3, a microtubule-associated protein1A/B light chain3, is involved in cytoplasmic lipid droplet formation. Biochem Biophys Res Commun.

[23] Kwanten WJ, Martinet W, Michielsen PP, Francque SM (2014). Role of autophagy in the pathophysiology of nonalcoholic fatty liver disease: a controversial issue. World J Gastroenterol.

[24] Singh R, Kaushik S, Wang Y, Xiang Y, Novak I, Komatsu M, Tanaka K, Cuervo AM, Czaja MJ (2009). Autophagy regulates lipid metabolism. Nature.

[25] Ma D, Molusky MM, Song J, Hu CR, Fang F, Rui C, Mathew AV, Pennathur S, Liu F, Cheng JX, Guan JL, Lin JD (2013). Autophagy deficiency by hepatic FIP200 deletion uncouples steatosis from liver injury in NAFLD. Mol Endocrinol.

[26] Wang SS, Chen YH, Chen N, Wang LJ, Chen DX, Weng HL, Dooley S, Ding HG (2017). Hydrogen sulfide promotes autophagy of hepatocellular carcinoma cells through the PI3K/Akt/mTOR signaling pathway. Cell Death Dis.

[27] Wang P, Guo QS, Wang ZW, Qian HX (2013). HBx induces HepG-2 cells autophagy through PI3K/Akt-mTOR pathway. Mol Cell Biochem.

[28] Fiorotto R, Raizner A, Morell CM, Torsello B, Scirpo R, Fabris L, Spirli C, Strazzabosco M (2013). Notch signaling regulates tubular morphogenesis during repair from biliary damage in mice. J Hepatol.

[29] Geisler F, Strazzabosco M (2015). Emerging roles of notch signaling in liver disease. Hepatology.

[30] Lu J, Xia Y, Chen K, Zheng Y, Wang J, Lu W, Yin Q, Wang F, Zhou Y, Guo C (2016). Oncogenic role of the notch pathway in primary liver cancer. Oncol Lett.

[31] Gao B, Bataller R (2011). Alcoholic liver disease: pathogenesis and new therapeutic targets. Gastroenterology.

[32] Powell EE, Jonsson JR, Clouston AD (2005). Steatosis: co-factor in other liver diseases. Hepatology.

[33] Watanabe H, Rose MT, Aso H (2011). Role of peripheral serotonin in glucose and lipid metabolism. Curr Opin Lipidol.

[34] Martin AM, Young RL, Leong L, Rogers GB, Spencer NJ, Jessup CF, Keating DJ (2017). The diverse metabolic roles of peripheral serotonin. Endocrinology.

[35] Namkung J, Kim H, Park S (2015). Peripheral Serotonin. a new player in systemic energy homeostasis. Mol Cells.

[36] Oh CM, Namkung J, Go Y, Shong KE, Kim K, Kim H, Park BY, Lee HW, Jeon YH, Song J, Shong M, Yadav VK, Karsenty G, Kajimura S, Lee IK, Park S, Kim H (2015). Regulation of systemic energy homeostasis by serotonin in adipose tissues. Nat Commun.

[37] Langeslag S, van der Veen F, Fekkes D (2012). Blood levels of serotonin are differentially affected by romantic love in men and women. J Psychophysiol.

[38] Kumar AM, Weiss S, Fernandez JB, Cruess D, Eisdorfer C (1998). Peripheral serotonin levels in women: role of aging and ethnicity. Gerontology.

[39] Lowery CL, Elliott C, Cooper A, Hadden C, Sonon RN, Azadi P, Williams DK, Marsh JD, Woulfe DS, Kilic F (2017). Cigarette smoking-associated alterations in serotonin/adrenalin signaling pathways of platelets. J Am Heart Assoc.

[40] Vikenes K, Farstad M, Nordrehaug JE (1999). Serotonin is associated with coronary artery disease and cardiac events. Circulation.

[41] Selim AM, Sarswat N, Kelesidis I, Iqbal M, Chandra R, Zolty R (2017). Plasma serotonin in heart failure: possible marker and potential treatment target. Heart Lung Circ.

[42] Pietraszek MH, Urano T, Sumioshi K, Serizawa K, Takahashi S, Takada Y, Takada A (1991). Alcohol-induced depression: involvement of serotonin. Alcohol Alcohol.

[43] Morgan CJ, Badawy AA (2001). Alcohol-induced euphoria: exclusion of serotonin. Alcohol Alcohol.

[44] Kumar AM, Sevush S, Kumar M, Ruiz J, Eisdorfer C (1995). Peripheral serotonin in Alzheimer's disease. Neuropsychobiology.

[45] Shu B, Zhai M, Miao X, He C, Deng C, Fang Y, Luo M, Liu L, Liu S (2018). Serotonin and YAP/VGLL4 balance correlated with progression and poor prognosis of hepatocellular carcinoma. Sci Rep.

[46] Abdel-Razik A, Elhelaly R, Elzehery R, El-Diasty A, Abed S, Elhammady D, Tawfik A (2016). Could serotonin be a potential marker for hepatocellular carcinoma? A prospective single-center observational study. Eur J Gastroenterol Hepatol.

[47] White E (2015). The role for autophagy in cancer. J Clin Invest.

[48] Takamura A, Komatsu M, Hara T, Sakamoto A, Kishi C, Waguri S, Eishi Y, Hino O, Tanaka K, Mizushima N (2011). Autophagy-deficient mice develop multiple liver tumors. Genes Dev.

[49] Trulson ME, Sampson HW (1986). Ultrastructural changes of the liver following L-tryptophan ingestion in rats. J Nutr.

[50] Matthies DL, Jacobs FA (1993). Rat liver is not damaged by high dose tryptophan treatment. J Nutr.

[51] Haub S, Kanuri G, Volynets V, Brune T, Bischoff SC, Bergheim I (2010). Serotonin reuptake transporter (SERT) plays a critical role in the onset of fructose-induced hepatic steatosis in mice. Am J Physiol Gastrointest Liver Physiol.

[52] Chen X, Margolis KJ, Gershon MD, Schwartz GJ, Sze JY (2012). Reduced serotonin reuptake transporter (SERT) function causes insulin resistance and hepatic steatosis independent of food intake. PLoS One.

[53] Chang AS, Chang SM, Starnes DM, Schroeter S, Bauman AL, Blakely RD (1996). Cloning and expression of the mouse serotonin transporter. Brain Res Mol Brain Res.

[54] Ramamoorthy S, Bauman AL, Moore KR, Han H, Yang-Feng T, Chang AS, Ganapathy V, Blakely RD (1993). Antidepressant- and cocaine-sensitive human serotonin transporter: molecular cloning, expression, and chromosomal localization. Proc Natl Acad Sci U S A.

[55] The Human Protein atlas. https://www.proteinatlas.org/ENSG00000108576-SLC6A4/cell. Accessed 20 Aug 2018.

