# Supplementary material for: Serotonin induced hepatic steatosis is associated with modulation of autophagy and notch signaling pathway
Source: Cell Commun Signal. 2018 Nov 8;16:78. doi: 10.1186/s12964-018-0282-6 (PMC6225666; doi:10.1186/s12964-018-0282-6)
Supplement: Supplementary file 1 — Table S1. The following primer sets were used for analysis of fatty acid/lipid metabolic gene expression and serotonin receptor (5-HTrs) expression using RT/qPCR. (DOCX 15 kb) [file 12964_2018_282_MOESM1_ESM.docx]

**Table S1**

Set of primers used for analysis of lipid and fitty acid metabolic gene expression and serotonin receptor expression in RT/qPCR.

| FAS FP: 5’-CTAGGTTTGATGCCTCCTTCTT-3’  FAS RP: 5’-GATGGCTTCATAGGTGACTTCC-3’  PPARγ FP: 5’-CTCAAACGAGAGTCAGCCTTTA-3’  PPARγ RP: 5’-GTGGGAGTGGTCTTCCATTAC-3’  SCD1 FP: 5’-CTCTTTCTGCTCTGCCATCTT-3’  SCD1 RP: 5’-CCCGACTTCACCTCCTTAAATC-3’  L-FABP FP: 5’-GGAATGTGAGCTGGAGACAA-3’  L-FABP RP: 5’-AGTTCGGTCACAGACTTGATG  SREBP1 FP: 5’-GAGCCATGGATTGCACTTTC-3’  SREBP1 RP: 5’-AGCATAGGGTGGGTCAAATAG-3’  HNF4α FP: 5’-GGAGAGGACAAGATGGGTAAAC-3’  HNF4α RP: 5’-TAAGACAGTGCCTGGGAGTA-3’  ACC FP: 5’-GCAGGTCACACGTCTCTTTAT-3’  ACC RP: 5’-CCAGCCTGTCATCCTCAATATC-3’  5-HTR1A FP: 5’- CATCTCGCTCACTTGGCTTAT-3’  5-HTR1A RP:5’- CGACTCTCCATTCACACTCTTC-3’  5- HTR1B FP: 5’- GAATCCGGATCTCCTGTGTATG-3’  5- HTR1B RP: 5’-GGTTGATGAGGGAGTTGAGATAG-3’  5-HTR2A FP: 5’- CTGTGTCAGGATTGAGGATGAA-3’  5-HTR2A RP: 5’- CCTCCCAAAGTGCTAGGATTAC-3’  5-HTR7 FP: 5’- GGCAGAGTCGAGAAAGTTGT-3’  5-HTR7 RP: 5’- CCATCCAAAGAGTGGAGGTAAG-3’  5-HTR4 FP: 5’- GTGAGTTCTGAGGAGGGTTTC-3’  5-HTR4 RP: 5’- GGCAGCAGATGGCGTAATA-3’  GAPDH FP: 5’- CCACCCAGAAGACTGTGGAT -3’  GAPDH RP: 5’- GTTGAAGTCAGAGGAGACCACC -3’ |
| --- |
