# Supplementary figures and images for: Serotonin induced hepatic steatosis is associated with modulation of autophagy and notch signaling pathway
Source: Cell Commun Signal. 2018 Nov 8;16:78. doi: 10.1186/s12964-018-0282-6 (PMC6225666; doi:10.1186/s12964-018-0282-6)

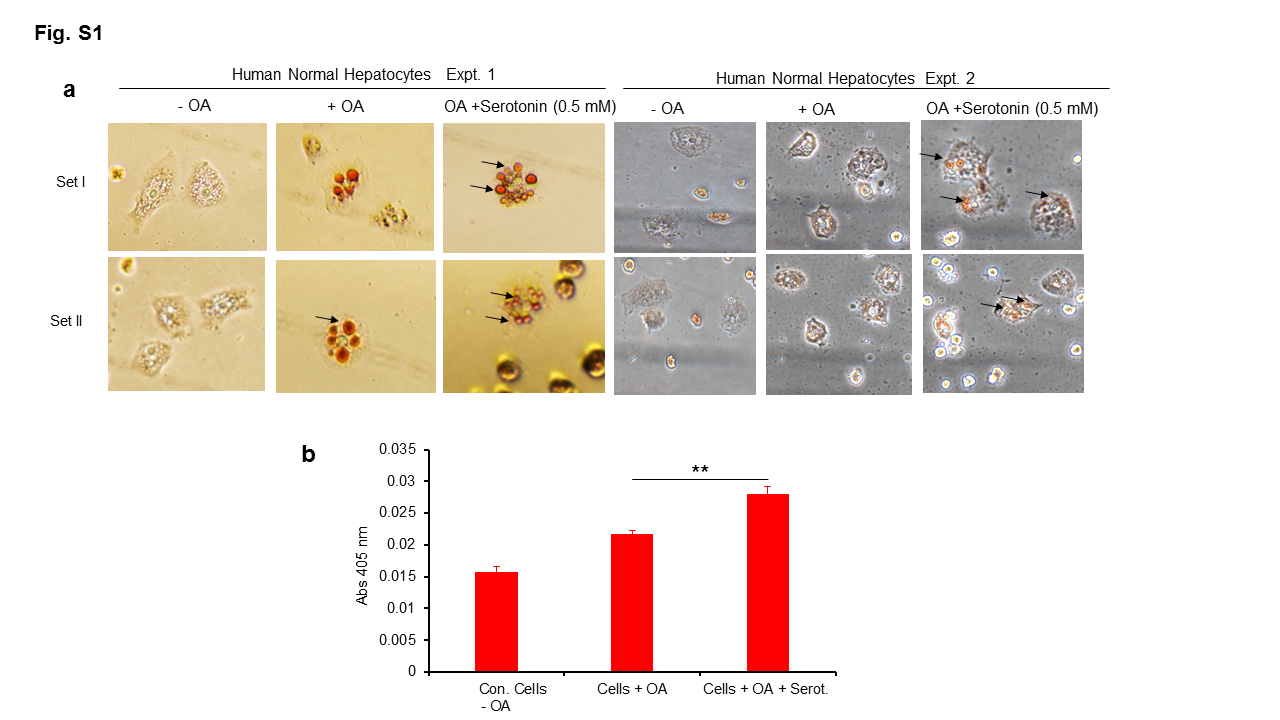

Supplement: Supplementary file 2 — Figure S1. Serotonin induces cell steatosis in normal human hepatocytes. (a) Normal human hepatocytes were grown in 6-well plates coated with Collagen 1 (Gibco) for 24 h and treated with vehicle or 100 μM oleic acid (OA) alone, or OA and serotonin (0.5 mM) for 24 h. Cells were fixed, stained with Oil Red O stain only, and observed under using light microscopy and photographed as described earlier. (b) After Oil Red O staining, the same cells were lysed in cell lysis buffer (100 μl). Oil Red O stain released from steatotic cells was then transferred to another 96-well plate and the absorbance at 405 nm was measured using the Fluostar Omega plate reader as described previously [19]. **p < 0.01 compared to OA treated cells. (TIF 566 kb) [file 12964_2018_282_MOESM2_ESM.tif]

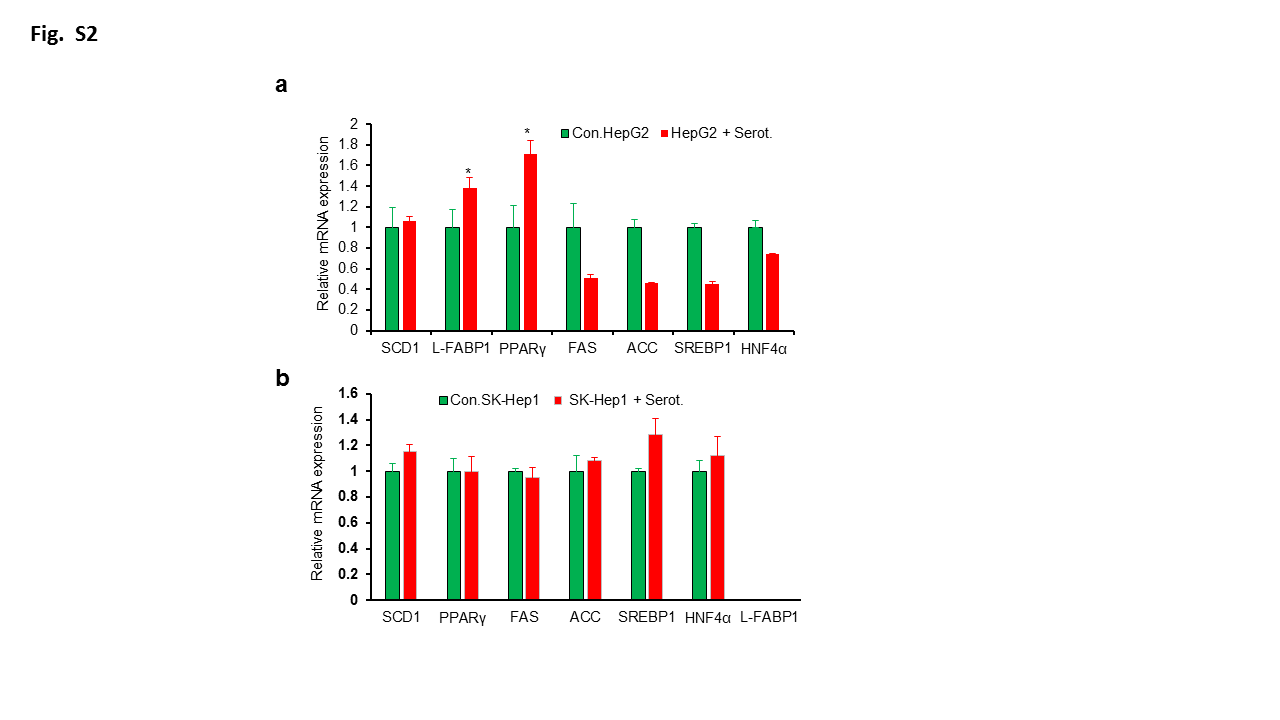

Supplement: Supplementary file 3 — Figure S2. Serotonin modulates the expression of fatty acid and lipid metabolic genes in HepG2 and SK-Hep1 cells. (a) HepG2 and (b) SK-Hep1 cells were grown in 6-well plates and treated with 0.5 mM of serotonin for 30 h. Total RNA was isolated from untreated and serotonin treated cells using TRIZOL reagent, and the expression of fatty acid and lipid metabolic gene expression was analyzed by RT/qPCR as described in the materials and methods sections. Data are expressed as the mean ± S.D. *p < 0.1 compared to untreated control cells. (TIF 83 kb) [file 12964_2018_282_MOESM3_ESM.tif]

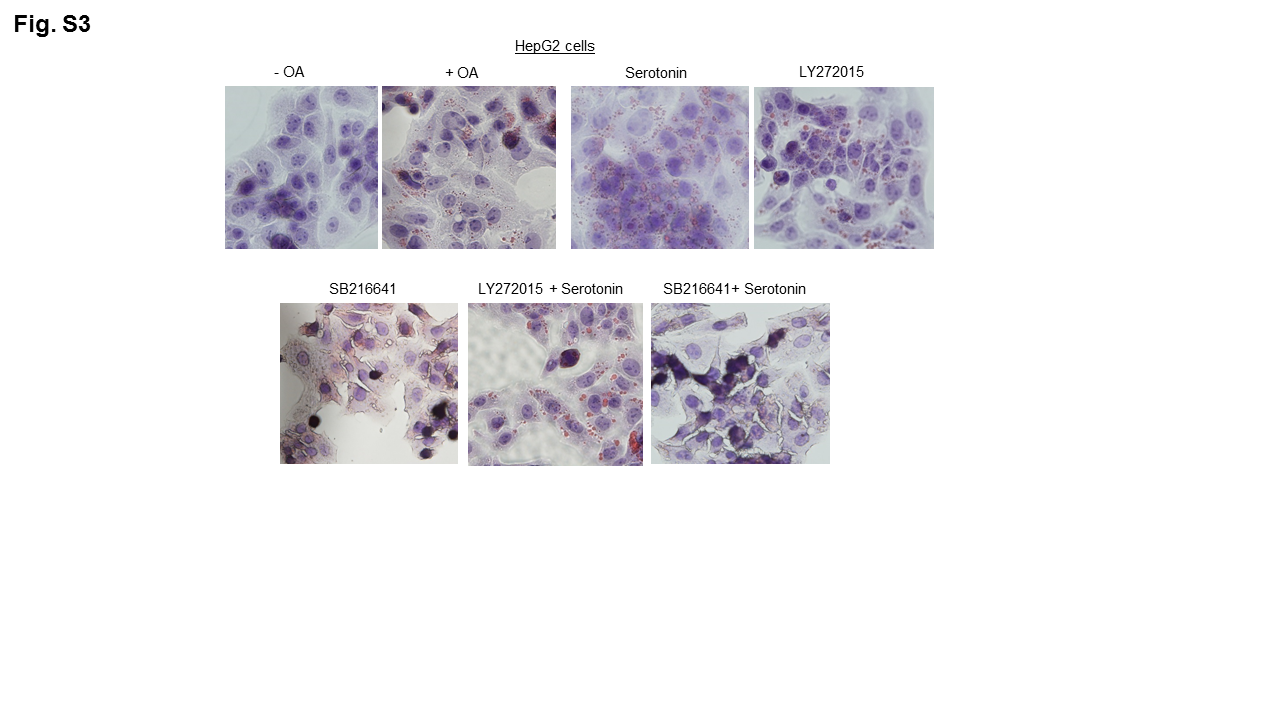

Supplement: Supplementary file 4 — Figure S3. Effect of serotonin receptor antagonists on HepG2 cell steatosis. HepG2 cells were grown on coverslips in 6-well plates and treated with indicated concentrations of serotonin, LY272015 or SB216641 alone, or in combination, as indicated. Cells were further treated with 100 μM oleic acid for an additional 24 h. Cells were fixed, stained with Oil Red O stain, and observed under a light microscope and photographed. (TIF 508 kb) [file 12964_2018_282_MOESM4_ESM.tif]

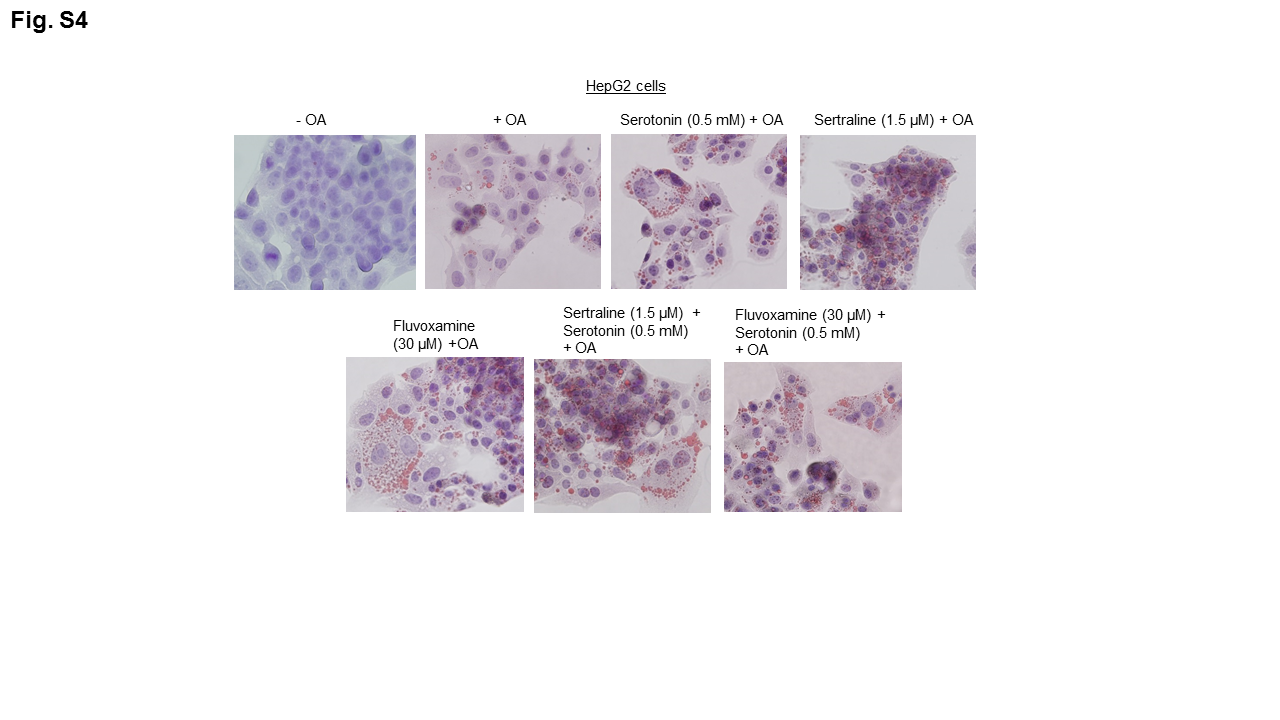

Supplement: Supplementary file 5 — Figure S4. Effect of serotonin re-uptake inhibitors (SSRIs) on HepG2 cell steatosis. HepG2 cells were grown on coverslips in 6-well plates and treated with serotonin or serotonin re-uptake inhibitors (SSRIs), sertraline and fluvoxamine, alone for 30 h, or pretreated with sertraline and fluvoxamine for 8 h followed by serotonin treatment for 24 h in the presence of SSRIs as indicated. Cells were further treated with vehicle alone or 100 μM oleic acid for additional 18 h. Cells were stained with Oil Red O stain and observed under a light microscope and photographed as described earlier. (TIF 450 kb) [file 12964_2018_282_MOESM5_ESM.tif]

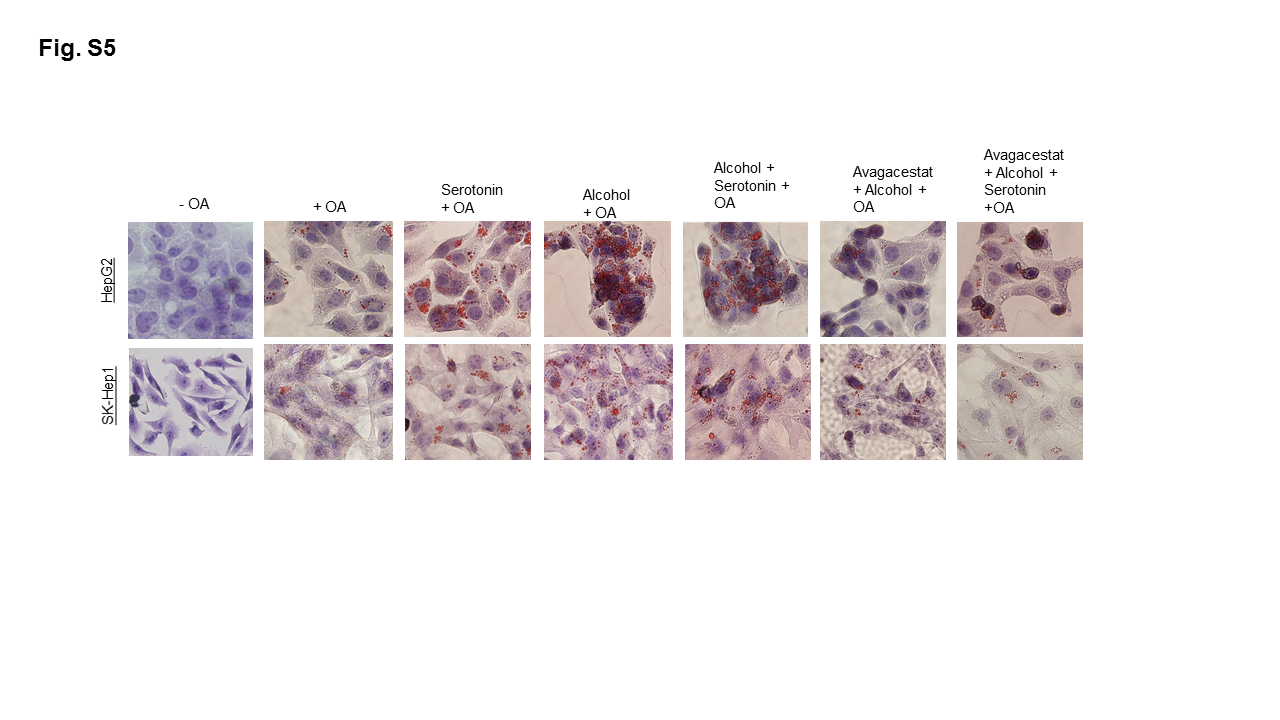

Supplement: Supplementary file 6 — Figure S5. Effect of EtOH on liver cancer cell steatosis. HepG2 and SK-Hep1 cells were grown on coverslips in 6-well plates and treated with serotonin (0.5 mM), EtOH (50 mM), or in combination with Notch inhibitor avagacestat (2 μM) as indicated for 24 h. Cells were further treated with vehicle alone or 100 μM oleic acid and stained with Oil Red O. Cells were stained with Oil Red O and observed under a light microscope and photographed. (TIF 587 kb) [file 12964_2018_282_MOESM6_ESM.tif]
